# Quantifying the impacts of land cover change on gross primary productivity globally

**DOI:** 10.1038/s41598-022-23120-0

**Published:** 2022-11-01

**Authors:** Andreas Krause, Phillip Papastefanou, Konstantin Gregor, Lucia S. Layritz, Christian S. Zang, Allan Buras, Xing Li, Jingfeng Xiao, Anja Rammig

**Affiliations:** 1 TUM School of Life Sciences Weihenstephan, Technical University of Munich, Hans-Carl-Von-Carlowitz-Platz 2, 85354 Freising, Germany; 2 Weihenstephan-Triesdorf University of Applied Sciences, Hans-Carl-Von-Carlowitz-Platz 3, 85354 Freising, Germany; 3 Research Institute of Agriculture and Life Sciences, Seoul National University, Seoul, South Korea; 4 Earth Systems Research Center, Institute for the Study of Earth, Oceans, and Space, University of New Hampshire, Durham, NH 03824 USA

**Keywords:** Ecological modelling, Carbon cycle, Climate and Earth system modelling, Climate-change mitigation

## Abstract

Historically, humans have cleared many forests for agriculture. While this substantially reduced ecosystem carbon storage, the impacts of these land cover changes on terrestrial gross primary productivity (GPP) have not been adequately resolved yet. Here, we combine high-resolution datasets of satellite-derived GPP and environmental predictor variables to estimate the potential GPP of forests, grasslands, and croplands around the globe. With a mean GPP of 2.0 kg C m^−2^ yr^−1^ forests represent the most productive land cover on two thirds of the total area suitable for any of these land cover types, while grasslands and croplands on average reach 1.5 and 1.8 kg C m^−2^ yr^−1^, respectively. Combining our potential GPP maps with a historical land-use reconstruction indicates a 4.4% reduction in global GPP from agricultural expansion. This land-use-induced GPP reduction is amplified in some future scenarios as a result of ongoing deforestation (e.g., the large-scale bioenergy scenario SSP4-3.4) but partly reversed in other scenarios (e.g., the sustainability scenario SSP1-1.9) due to agricultural abandonment. Comparing our results to simulations from state-of-the-art Earth System Models, we find that all investigated models deviate substantially from our estimates and from each other. Our maps could be used as a benchmark to reduce this inconsistency, thereby improving projections of land-based climate mitigation potentials.

## Introduction

Terrestrial ecosystems exchange large amounts of carbon with the atmosphere and thus play a crucial role in the global carbon cycle. Gross primary productivity (GPP), the amount of carbon fixed via photosynthesis, is the largest carbon flux between land and atmosphere (~ 130 Gt C yr^−1^)^1^. Around half of the GPP is quickly released back to the atmosphere as autotrophic respiration while the remainder, net primary productivity (NPP) is available for biomass production. GPP and NPP thus co-determine not only the carbon uptake potential of ecosystems but also other ecosystem services such as the supply of wood products, food, fodder, and bioenergy.

Presently, vegetation and soils absorb around 30% of anthropogenic CO_2_ emissions^2^, thereby slowing down the increase in atmospheric CO_2_ and mitigating climate change. However, over most of the Holocene, the terrestrial biosphere acted as a net carbon source as humans gradually converted more than one third of the global land area into croplands or managed grasslands^3^, thereby reducing total biomass by around 260 Gt C^4^ and soil carbon by around 116 Gt C^5^. Reversing these carbon losses and enhancing terrestrial carbon storage via forest protection and expansion are thus increasingly considered as effective measures to achieve the targets of the Paris Agreement^6^. Nevertheless, while it is clear that forests store more carbon than agricultural land^7^, the question whether they are also superior in terms of productivity (GPP and NPP) has so far received less attention and is much more uncertain.

The impacts of land cover changes on the terrestrial carbon cycle have so far mainly been estimated using process-based ecosystem models such as Dynamic Global Vegetation Models or Earth System Models (ESMs)^2,8–14^. However, there is a large spread in simulated global productivity in these models^15^ and no agreement concerning the question of how land cover changes affect ecosystem productivity^8,11,16^. In fact, the large spread in simulated carbon stock changes in response to deforestation or reforestation across ecosystem models has largely been attributed to the uncertainty regarding the magnitude and direction of change in productivity associated with land cover change^8,11^. For instance, LPJmL simulates large increases in ecosystem productivity following reforestation, while ORCHIDEE leads to productivity reductions^11^. This is likely related to differences in how the models incorporate land-use changes and which natural and management processes are considered (e.g., LPJmL accounted for nitrogen fertilization and limitation, while ORCHIDEE did not). Maps of the potential productivity of different land cover types would therefore provide valuable benchmarks for model evaluation and help to narrow down the uncertainty concerning the impacts of land cover changes on carbon storage. This is urgently needed to assess the plausibility of land-based climate mitigation scenarios given our limited understanding of the terrestrial carbon cycle^2,9^ and increasing evidence that even relatively small levels of climate change might have dramatic impacts on ecosystems and societies^6^.

Besides ecosystem modelling, large-scale GPP patterns can also be investigated via remote sensing. A major recent advancement is the measuring of Solar-Induced Chlorophyll Fluorescence (SIF) which is used to study photosynthesis^17,18^. Previous studies have reported either specific or universal SIF–GPP relationships across biomes using SIF from satellites and GPP from eddy covariance flux towers or gridded products^17–19^. The SIF–GPP relationship is affected by many factors such as differences in sun-target-sensor geometry, scale mismatch between satellite and tower footprint, biases in SIF retrievals and gridded GPP products^17,20^. Recently, the GOSIF GPP product was derived from the ensemble mean GPP of eight SIF–GPP relationships, which partly reduces uncertainty resulting from the variations of relationship across biomes^21^. The availability of SIF observations globally and the close relationship between SIF and GPP thus allow for an independent assessment of how land cover changes affect GPP in different regions around the world.

Here we estimate the productivity of different land cover types by combining the high-resolution GOSIF GPP product, land cover from the European Space Agency Climate Change Initiative (ESA-CCI)^22^, and 20 environmental predictor variables in a machine learning approach using Random Forests (RF) (see Supplementary Fig. S1). We produce global maps of the potential GPP of forests, grasslands, and croplands, which are subsequently analysed jointly with state-of-the-art land-use change reconstructions and scenarios^23^ to estimate associated impacts on GPP. We also investigate the robustness of our results by conducting a sensitivity analysis in which we test a range of alternative input datasets and algorithms. Furthermore, we compare our potential GPP estimates to ESM simulations from the 6th phase of the Coupled Model Intercomparison Project (CMIP6). We thereby address the following questions: (1) What is the potential GPP that forests, grasslands, and croplands can realize under identical environmental conditions? (2) What is the impact of land cover changes (both past and future) on global GPP? (3) Do state-of-the-art ESMs from CMIP6 agree on the simulated GPP of these land cover types and how do the simulations compare to our empirically derived estimate? Our study sheds light on a crucial, yet poorly constrained aspect of the terrestrial carbon cycle and its representation in the current generation of ESMs, potentially improving estimates of land cover change impacts on the carbon-climate system and the provisioning of ecosystem services.

## Results and discussion

### Forests are typically the most productive land cover type

**Figure 1:**
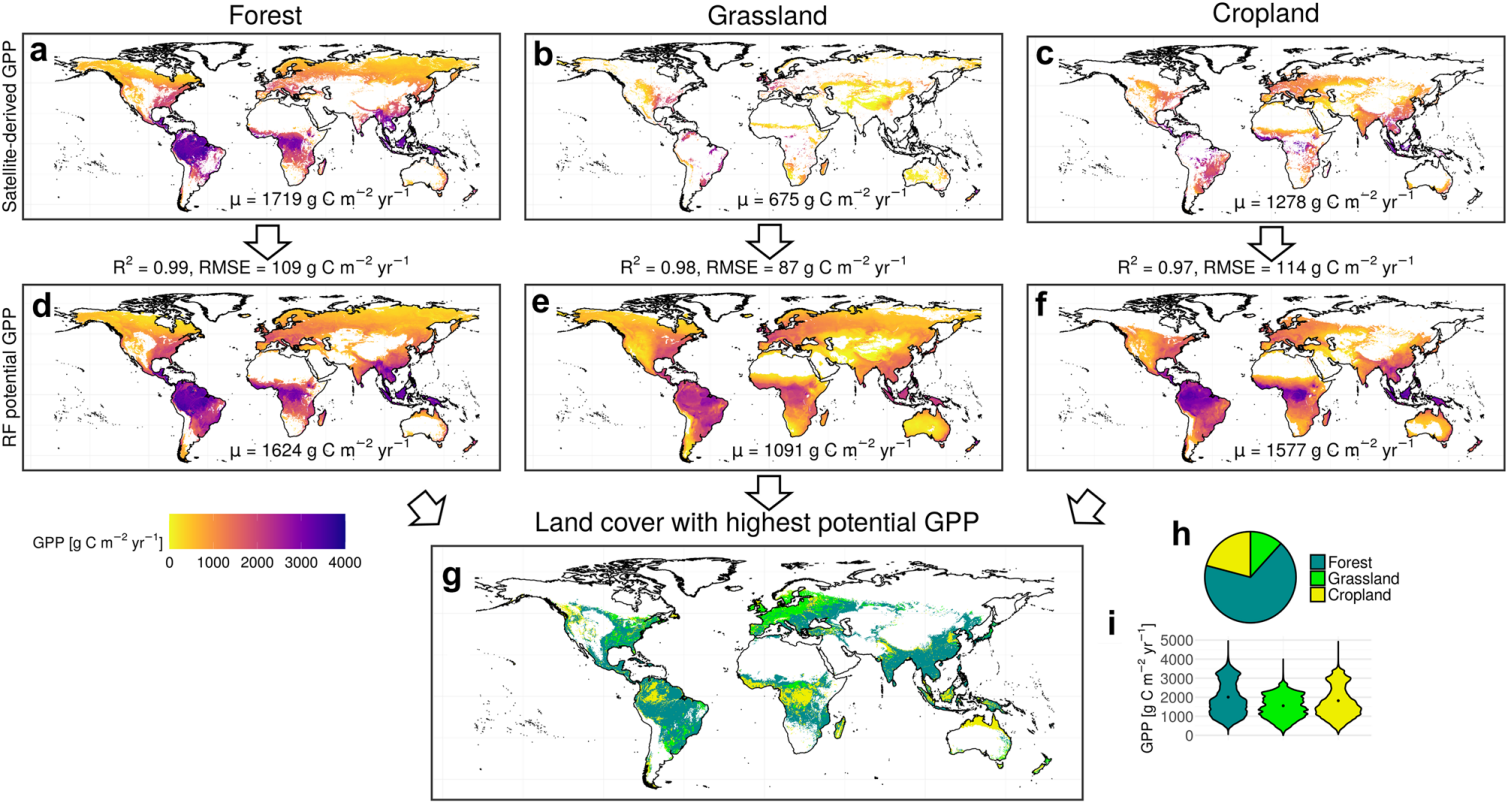
Maps of potential GPP for different land cover types derived from RF predictions. (**a**–**c**) Satellite-derived present-day GPP for forests (**a**), grasslands (**b**) and croplands (**c**) (i.e., the training data). (**d**–**f**) Potential GPP predicted by the RF algorithm. (**g**) Land cover with highest potential GPP according to (**d**–**f**). (**h**) Global fractions of the most productive land cover type. **i**, Potential GPP distribution across the total suitable area. R^2^ and RMSE values are computed on the out-of-bag testing data. The good model performance can partly be explained by the very large training data and to some degree by spatial autocorrelation^24^ (Supplementary Discussion 2 and Figs. S3 and S4). Global area-weighted GPP means are given by the numbers at the bottom of the maps. Grid cells where no forests exist today or potential forest cover (Supplementary Fig. S5) is < 36.3% (i.e., 5th percentile of all currently forested grid cells) or which are too cold or dry for grass/crop growth are removed from (**d**–**f**) and removed from (**g**) if unsuitable for at least one land cover type. Dots in (**i**) indicate area-weighted means. Maps were created using R version 4.1.0 (https://cran.r-project.org/)^25^.

Remotely-sensed patterns of present-day GPP for each considered land cover type (forest, grassland, cropland) can be reproduced by the RF models with high accuracy and extrapolated into new areas (Fig. 1a–f). In the subsequent analyses, we focus on suitable areas where environmental conditions would allow the existence of all three land cover classes (Fig. 1g). According to our RF predictions, potential forest GPP exceeds the potential GPP of grasslands and croplands on 67% of the total suitable area, especially in Southeast Asia, large parts of South America, southeastern Europe, and African dry forests (Fig. 1g+h). Croplands are most productive on 21% of the suitable area, mostly in Central Africa, Indonesia and northern Australia, western North America, and parts of the Amazon, while grasslands are most productive in large parts of Central Europe and the Eastern US (12%) (even though by a small margin, also see continental mean values in Supplementary Table 1). Many of the subtropical and temperate areas suitable for all three land cover types are presently used for agriculture while in the inner tropics native forests are still prevalent. Mean potential forest GPP in suitable areas is 2.0 kg C m^−2^ yr^−1^, while grassland and cropland potential GPP is 1.5 and 1.8 kg C m^−2^ yr^−1^, respectively (Fig. 1i). This implies that on average grasslands and croplands reach only 77 and 91% of forest productivity, respectively, the former being well in line with Haberl et al.^13^ who assumed a 22% NPP reduction when converting forests to grazing land based on ecosystem modelling and site data. These findings are qualitatively consistent across alternative input datasets and machine learning algorithms even though somewhat sensitive to the underlying input land cover map (see Supplementary Discussion 1 and Fig. S2).

### Historical and future productivity changes arising from land cover changes

**Figure 2:**
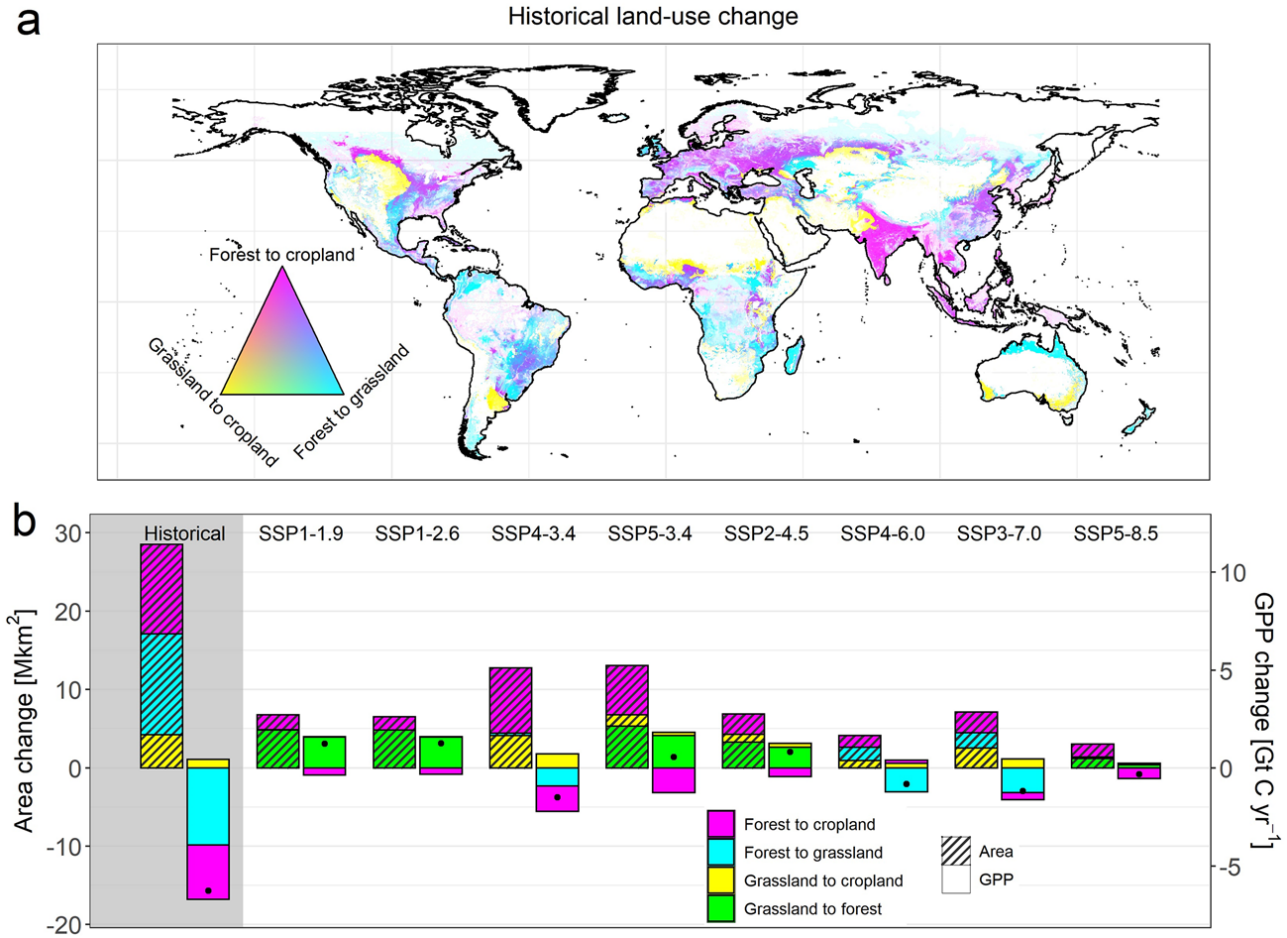
Land cover change impacts on GPP. (**a**) Map of historical (until year 2015) land cover transitions based on LUH2 agricultural areas and potential forest cover calculated from ref.^31^. Shading indicates the converted fraction of a grid cell. (**b**) Global net land cover transitions as well as associated impacts on global GPP for the historical period (until year 2015, grey background) and future projections (2015–2100). The net impacts of all land cover transitions on global GPP are indicated by dots. The figure was created using R version 4.1.0 (https://cran.r-project.org/)^25^.

To investigate the impacts of anthropogenic land cover changes on global GPP we combine our derived potential GPP maps with maps of historical agricultural expansion and future land-use changes as provided by the second phase of the Land-Use Harmonization Project (LUH2)^23^. According to this approach, since the early Holocene humans have converted around 1.1 Mkm^2^ of forests into croplands and another 1.3 Mkm^2^ of forests into managed grassland, thereby reducing global GPP by 2.8 and 3.9 Gt C yr^−1^, respectively (Fig. 2). Cropland expansion in natural grasslands increased global GPP by 0.4 Gt C yr^−1^, resulting in a net GPP reduction of 6.3 Gt C yr^−1^. For comparison, present-day global GPP in our satellite-derived GPP dataset is 135.4 Gt C yr^−1^, implying a potential natural GPP of 141.7 Gt C yr^−1^ and a historical GPP reduction of 4.4% in response to deforestation and agricultural expansion. Our uncertainty analysis yields comparable reductions in ecosystem productivity for alternative potential productivity estimates (mean: 4.6%, range 2.5–6.0%; see Supplementary Fig. S6). These numbers are considerably smaller than the NPP reduction estimated in previous assessments based on MODIS NPP or ecosystem modelling combined with census data (7–10%)^13,26,27^. This is surprising given that the lower NPP/GPP ratio of forests compared to cultivated land^28^ should result in a larger GPP reduction in response to deforestation compared to NPP.

Future (2015–2100) land-cover related GPP changes are investigated for eight LUH2 scenarios, describing potential pathways in terms of social-economic development (Shared Socioeconomic Pathways; SSPs) combined with greenhouse gas trajectories (Representative Concentration Pathways, RCPs). In contrast to previous research^29^, the impacts of future land cover changes on productivity in our study are generally smaller than for the historical period. Some scenarios (SSP1-1.9, SSP1-2.6, SSP2-4.5, SSP5-3.4) assume large-scale forest restoration on managed grassland as a measure of climate mitigation, thereby increasing GPP by up to 1.3 Gt C yr^−1^ despite continued cropland expansion (Fig. 2b, Supplementary Fig. S7). The historical GPP reduction could thus partly be reversed in these scenarios. In contrast, other scenarios assume continued large-scale deforestation as a result of high population growth, animal-based diets, and low agricultural land intensification (SSP3-7.0) or bioenergy cultivation for climate mitigation (SSP4-3.4), thereby decreasing GPP further by up to 1.5 Gt C yr^−1^. However, it should be noted that second-generation bioenergy crops like *Miscanthus*, which are assumed in some of these scenarios to be planted at a large scale, might turn out to be more productive than conventional crops^30^. In addition, our potential GPP maps do not account for future productivity changes due to climate change and increasing atmospheric CO_2_ or changes in land management (e.g., fertilization).

### Theoretical maximum gross primary productivity

We also provide an estimate of the theoretical maximum GPP achievable if all land areas were converted to the most productive land cover type (Supplementary Fig. S8). In such case, global GPP would be 13.1 Gt C yr^−1^ higher than presently (or 6.8 Gt C yr^−1^ higher than under potential natural vegetation). Much of this increase comes from forest growth on current agricultural land, in particular in the tropics where the mean GPP-effectiveness of forest growth is about twice compared to temperate and boreal regions (Supplementary Fig. S9). However, there is also considerable potential for cropland expansion at other tropical locations and for grassland expansion in high latitudes. We want to emphasize that this represents a theoretical idea rather than a practical recommendation as such large-scale land cover conversions would dramatically impact ecosystem carbon storage (secondary forests require several centuries to achieve a carbon equilibrium^32^), food production patterns, biodiversity, and other ecosystem functions. In particular, old-growth forests require conservation as they do not only store large amounts of carbon but also provide many other ecosystem services and feedbacks with local climate, e.g. biophysical cooling and water recycling due to high evapotranspiration rates.

### Disagreement on the most productive land cover type across CMIP6 earth system models

**Figure 3:**
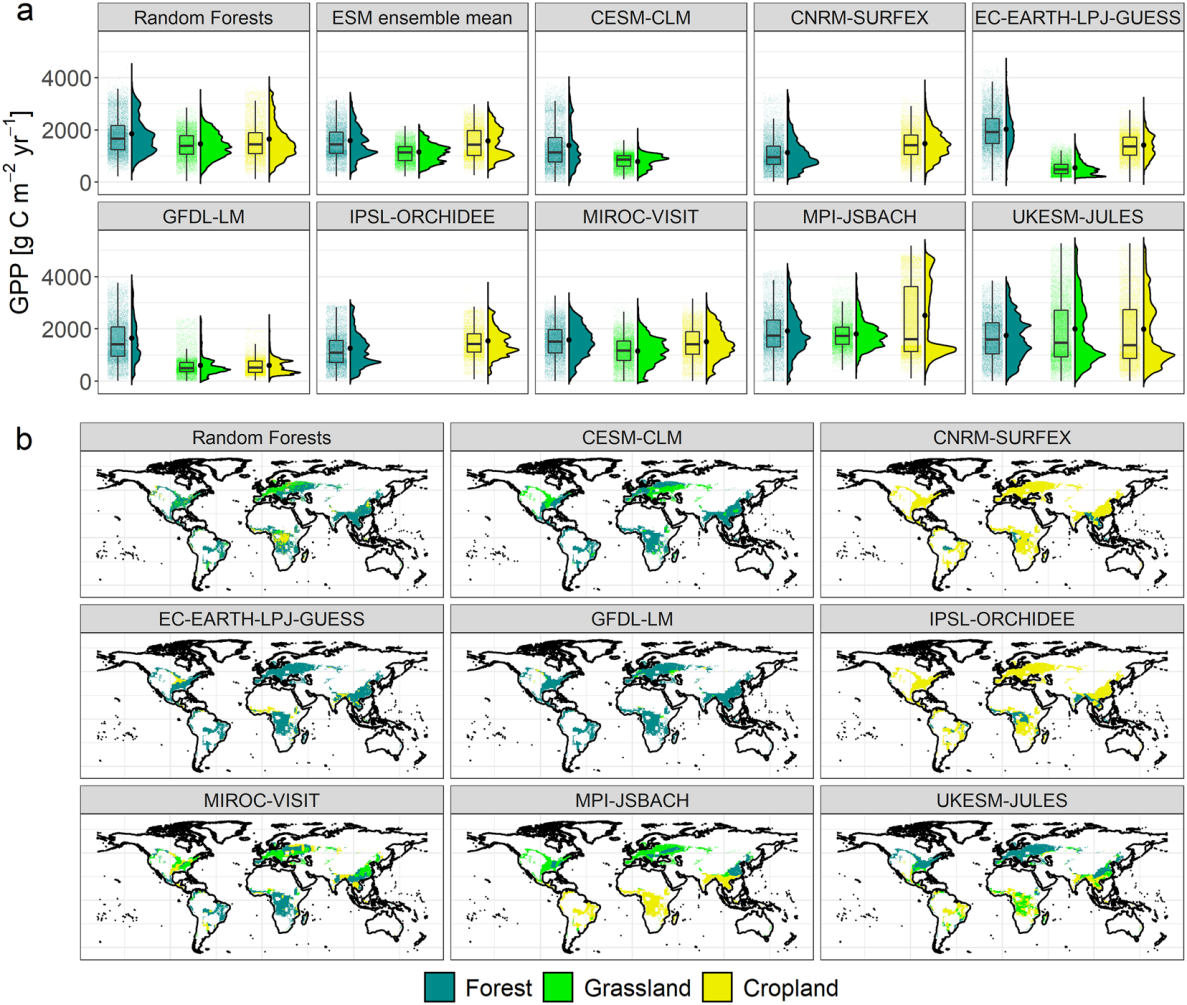
Comparison of our RF GPP predictions to CMIP6 ESM simulations. (**a**) Box and violin plots of GPP probability densities for different land cover types in our RF approach, eight ESMs and the ESM ensemble (2001–2014 mean values). The tiny coloured dots correspond to individual grid cells (subset of 1% randomly selected cells), the larger black dots indicate area-weighted means across all grid cells. (**b**) Maps of the most productive land cover type. ESM considerably vary in their spatial resolutions and simulated forest cover so we bilinear remapped the output to 0.05° resolution using Climate Data Operators^33^ and removed grid cells without any trees in at least one ESM, i.e., we compare the same area for all models. Maps of the most productive land cover type on the original ESM spatial resolution can be found in Supplementary Fig. S10. The figure was created using R version 4.1.0 (https://cran.r-project.org/)^25^.

We assess how well state-of-the-art ESMs capture present-day forest, grassland, and cropland GPP by comparing our potential GPP estimates to simulations from eight ESMs participating in CMIP6. For all land cover types, there are large deviations across models regarding mean GPP values as well as the distribution (Fig. 3a). Compared to our RF approach, mean forest, grassland, and cropland GPP in the ESM ensemble are underestimated by 14, 21, and 4%, respectively. There is little consistency between individual ESMs and our approach at the grid cell level even though the ESM ensemble mean performs quite well (R^2^: 0.43–0.69; Supplementary Table 2). Most importantly, ESMs differ in what they assume to be the most productive land cover. In three out of eight ESMs, forests are clearly the most productive land cover globally while grassland and/or cropland GPP is substantially underestimated compared to our RF approach (Fig. 3a). For the other ESMs, differences in mean GPP across land cover classes are smaller than in our RF approach and often the agricultural land cover classes are more productive than forests. The large spread in simulated agricultural GPP across ESMs is likely a result of differences in represented management processes (e.g., fertilization or crop sowing and harvest) and crop types as well as differences in grassland C3/C4 ratios simulated by the ESMs. Our analysis suggests that ESMs simulating grasslands and croplands to be more productive than forests (such as UKESM-JULES) will likely underestimate the (soil) carbon sequestration potential from avoided deforestation and reforestation, while ESMs simulating very low agricultural productivity (such as GFDL-LM) will likely overestimate their potential^11^. However, this also depends on the region where land-based mitigation takes place (spatial patterns of land cover changes in the LUH2 scenarios are shown in Supplementary Fig. S7). For instance, MPI-JSBACH and UKESM-JULES particularly overestimate grassland and cropland GPP in the tropics (Fig. 3b, Supplementary Fig. S10) but might be more reliable for the temperate zone. Overall, the large disagreement across ESMs and their deviations from our RF predictions imply an urgent need for model improvements to better represent the effects of land cover changes on terrestrial carbon cycling.

## Conclusion

In conclusion, we find that forests on average have a 29% and 10% larger GPP than grasslands and croplands, respectively. However, on one third of the total suitable are, agricultural land cover types would potentially be more productive. Intersecting our potential GPP maps with a land-use reconstruction yields a global GPP reduction in response to historical agricultural expansion of around 4.4%, which is smaller than the NPP reduction estimated in previous studies. Noteworthy, the reduction is relatively robust across a range of alternative approaches used to estimate potential GPP (mean: 4.6%; range 2.5–6.0%). Current land-use change projections range from continued deforestation to forest restoration, implying that the historical land-use-driven decline in GPP could either proceed or partly be reversed. However, current state-of-the-art ESMs diverge from our derived potential GPP estimates by either considerably underestimating or overestimating differences between forests and agricultural land cover types. These biases question the ability of these models to adequately simulate the impacts of anthropogenic land cover changes on the terrestrial carbon cycle and should be addressed in future model development. In particular, ESMs which overestimate forest productivity in comparison to agriculture will likely simulate too high atmospheric CO_2_ removal from reforestation measures. Our potential GPP maps can be used as a benchmark when evaluating terrestrial ecosystem models, thereby improving projections on ecosystem carbon cycling, natural climate solutions, crop yields, and other ecosystem services.

## Materials and methods

### GPP data

As our primary productivity product we used the GOSIF GPP dataset^21^ which utilizes the linear relationship between GPP and remotely-sensed SIF^34^. GOSIF GPP is available globally at 0.05° spatial resolution for the period 2000–2021, with the period 2001–2015 selected here (for a short summary of all datasets used in this study see Supplementary Table 3). GOSIF GPP is based on a gridded SIF product (GOSIF)^34^ which uses MODIS enhanced vegetation index and meteorological data for spatial scaling and is trained with millions of SIF observations from the coarser-resolution Orbiting Carbon Observatory-2^35^. The global coverage of GOSIF and the close relationship between SIF and GPP allow for an independent assessment of how land cover changes affect GPP in different regions around the world. For instance, SIF has been shown to capture the high GPP in the US Corn Belt derived from flux towers, while ecosystem models underestimated it^36^. While GPP can thus be empirically estimated from satellite SIF observations relatively reliably (even though some assumptions like the linear GPP–SIF relationship and its universality across biomes are still debated^20,37–39^), the calculation of NPP needs additional assumptions of autotrophic respiration. Therefore, we focused our study on GPP, but we included an NPP product in our uncertainty analysis. In addition to that, to account for the challenges and uncertainties in global GPP estimates we included four alternative GPP products in our sensitivity analysis (see below).

### Land cover mapping

Gridded land cover was derived from ESA-CCI^22^, a global land cover product designed for climate science. ESA-CCI is available at 300 m spatial resolution for the 1992–2020 period (https://cds.climate.copernicus.eu/). We first classified ESA-CCI land covers to forests, grasslands, and croplands according to IPCC classification: classes 50–100, 160, 170 forests (2,022,283 grid cells); classes 110 and 130 grasslands (509,297 grid cells); classes 10–40 croplands (950,025 grid cells). We focus on these three major land cover types to facilitate our analysis. We then converted the resulting map to 0.05° resolution by determining the prevalent (i.e., mode) land cover for each grid cell using the *aggregate* function from the *raster* package^40^ and only included grid cells in our training data in which the prevalent land cover was constant over the period 2001–2015. Other classes (e.g., cropland/natural vegetation mosaics) and grid cells where the land cover changed over the 2001–2015 period were not used for the RF training.

### Random forests

RF is a popular and efficient supervised machine learning technique which can be applied for classification and regression problems^41^. While complex, it is still easier to interpret compared to other machine learning methods such as Artificial Neural Networks. It has recently been applied to a wide range of ecological research questions, including the prediction of food^42^ and bioenergy^43^ crop yields, potential natural vegetation^31^, forest aboveground biomass^44^, soil respiration^45^, and soil carbon emissions from land-use change^5^ and is thus well suited for our approach. The “Forests” refer to a number of individual decision trees. For each tree, a random sample of the training data is selected and split multiple times based on a random subset of variables from which the one minimizing the weighted variance is selected by the algorithm. Model performance is computed directly on out-of-bag (OOB) data which is randomly omitted from the training data (36.8% of all grid cells). When RF is applied to new data, a weighted prediction of each individual decision tree contributes to the overall prediction. Variance in the individual trees, e.g., by selecting random subsets of the observations and random variables at each node improves the overall RF predictive skill. Model training and prediction were done using the R *ranger* package^46^. After initial testing (see Supplementary Fig. S11) we decided to set the number of individual decision trees to 800 and the number of variables to possibly split at in each node to 10. As the good evaluation measures of RF algorithms can be related to spatial autocorrelation^24^ we also tested a coordinate-only model and performed a leave-one-out cross validation including spatial buffers (Supplementary Discussion 2, Supplementary Fig. S3). Due to the large computational effort we reduced the number of decision trees to 100 for the buffered leave-one-out cross validation.

### Predictor variables

We predicted forest, grassland, and cropland potential GPP using the following 20 predictor variables in our RF algorithm: mean annual surface temperature (Tmean), mean diurnal temperature range (Tdiurnal), temperature seasonality (Tseason; standard deviation), minimum temperature of the coldest month (Tmin), annual temperature range (Tannual), mean temperature of the warmest quarter (Twarmest), mean annual precipitation (Pmean), precipitation seasonality (Pseason; coefficient of variation), precipitation of the wettest quarter (Pwettest), precipitation of the driest quarter (Pdriest), precipitation of the warmest quarter (Pwarmest), mean annual solar radiation (SR), growing degree days (GDD), relative humidity (RH), soil clay content (Clay), elevation (EL), nitrogen deposition (Ndep), nitrogen fertilization (NF), pesticide application (Pest), and gross domestic product (GDP; a proxy for agricultural management input other than NF and Pest). Overall Tmean, Tannual, and Pmean were the most important predictor variables (see Supplementary Discussion 3 and Fig. S12). We also tested other predictors (including additional bioclimatic variables, soil pH, irrigation, or phosphate fertilization) but found only negligible improvements in RF evaluation metrics and hence decided to restrict our analysis to the 20 predictors mentioned above.

Climate variables were taken from the CHELSA dataset^47,48^, remapped to 0.05° spatial resolution using the *aggregate* function from the *raster* package^40^. To only include years overlapping with our GPP data we used the CHELSA time-series data for the 2001–2013 period if available and 1979–2013 climatologies elsewise. Clay was derived from the Regridded Harmonized World Soil Database v1.2^49^. Ndep was taken from ISIMIP2b^50^, bilinear remapped from 0.5° to 0.05° spatial resolution using Climate Data Operators^33^. Elevation was obtained from WorldClim^51^. NF and Pest were derived from country-specific FAO data (e.g., https://ourworldindata.org/grapher/pesticide-use-per-hectare-of-cropland), i.e., we used the same value for all grid cells in a country. GDP was obtained from ref.^52^.

### Suitable area

For the comparison of potential forest, grassland, and cropland GPP in Fig. 1g–i we only included grid cells suitable for all three land cover types. For forests, we assumed forest cover possible if the grid cell is currently forested (e.g., all grid cells of our forest training data) or if the potential natural forest cover exceeds 36.3%. This threshold represents the 5th percentile of all currently forested grid cells. Potential natural forest cover was derived from a potential natural vegetation map, available for 20 biomes at 0.00833° spatial resolution^31^. To convert these biomes into potential natural forest cover we assumed 100% forest cover for the ten forest biomes and 30% forest cover for tropical savannah. Other biomes were not considered. We then aggregated the map to 0.05° spatial resolution by computing the mean of 36 grid cells using the *aggregate* function form the *raster* package^40^ (see Supplementary Fig. S5 for the resulting map). For grasslands and croplands, we computed the 5th percentile of Tmean and Pmean in the training data (− 9.9 °C and 165 mm for grasslands and 2.7 °C and 295 mm for croplands, respectively) and removed all grid cells below those thresholds, assuming these areas to be too cold or too dry for the respective land cover type. Finally, we calculated the land cover with the highest potential GPP for all overlapping grid cells.

### Sensitivity analysis

To explore the sensitivity and uncertainty of our RF approach we repeated our prediction using different input datasets, potential forest cover, and machine-learning approaches. The importance of the underlying potential forest map was estimated by replacing our potential forest map (Supplementary Fig. S5) by the LUH2 potential forest map (Supplementary Fig. S13)^23^. To explore the dependency on the land cover product we repeated our RF prediction using the spatially aggregated MODIS land cover map (MCD12C1; IGBP scheme), available at 0.05° spatial resolution^53^. We classified grid cells of classes 1, 2, 3, 4, 5, (all forests), 8 (woody savannahs) and 9 (savannahs) as forest. Classes 8 and 9 were included in forest because otherwise forest cover would be underestimated in the temperate and boreal zone. Class 10 was classified as grassland and class 12 as cropland. A comparison of ESA-CCI with MODIS reveals a substantially larger cropland area in ECA-CCI but a smaller grassland area (Supplementary Fig. S14).

The sensitivity to the climate product was tested by repeating our analysis using predictor variables from the WorldClim climatologies (1970–2000)^51^, aggregated from 30 s to 0.05° spatial resolution using the *aggregate* function from the *raster* package^40^. In contrast to CHELSA, growing degree days and relative humidity were not available from WorldClim but we included water vapour pressure as additional predictor.

We also tested four alternative global GPP products. The vegetation photosynthesis model (VPM) product, available for the period of interest at 0.05° spatial resolution, is based on improved light use efficiency theory and is driven by remotely sensed datasets and reanalysis climate data and land cover classification which also distinguishes C3 vs. C4 photosynthesis pathways^54^. The second product is derived from remote sensing considering radiation and canopy conductance limitations on GPP and is available at 0.05° resolution for the 2001–2012 period^55^. Land cover is not an input variable. The third product, FLUXCOM, uses machine learning to scale FLUXNET site GPP to the globe^56,57^. FLUXCOM is available at 0.0833° resolution and was conservative remapped to 0.05° using Climate Data Operators^33^ meaning that the GPP of different land cover types might be mixed in regions with heterogeneous land cover patterns. The forth product is the MODIS MOD17A3 GPP product^58^, available for the 2001–2013 period and aggregated to 0.05° resolution using the *raster* package^40^. It is derived from meteorological data, fraction of absorbed photosynthetic active radiation/leaf area index, and land cover. As there is also a MOD17A3 NPP product available we additionally conducted a prediction for potential NPP. The MOD17A3 NPP product is calculated as GPP minus maintenance and growth respiration estimated from allometric relationships linking daily biomass and annual growth of plant tissues to leaf area index^58^. This leads to additional uncertainty compared to the MOD17A3 GPP product.

To test the effect of an alternative RF algorithm we repeated our prediction with the RF algorithm from the Python *scikit-learn* library^59^ using the same number of decision trees (800). Additionally, we tested another machine-learning technique, a deep neural network (DNN), using the *PyTorch* library^60^. We selected 10 linear layers with 5 times alternating 128 and 256 nodes and a sigmoid output function. All layers were connected using the rectified linear unit activation function. We used the *adamW* optimizer with 0.0003 learning rate and 2000 epochs of training. To prevent overfitting, we included a 10% dropout after the 7th layer. Lastly, we included a very simple estimate of the most productive land cover based on the nearest neighbour using *scikit-learn’s BallTree* implementation together with the *Haversine* formula. For each grid cell we searched for the nearest forest, grassland, and cropland grid cell and assigned the respective GPP also to this grid cell. We thus assumed that environmental conditions are more or less identical in these grid cells, which might be a reasonable assumption for many locations but less reliable in complex terrain or in large homogeneous regions like the central Amazon rainforest where the nearest cropland/grassland grid cell might be located far away.

### Land-use change scenarios

To estimate the effects of historical and potential future land cover changes on global GPP we applied LUH2 scenarios^23^ which also serve as input data for ESMs participating in CMIP6. Land-use changes over the historical period are based on the HYDE reconstruction^3^, while future projections were developed by different Integrated Assessment Models combining various assumptions of socio-economic behaviour (SSPs) with climate mitigation targets (RCPs). Annual fractions for the two land cover classes cropland (sum of 5 crop types) and managed grassland (sum of pasture and rangeland) were available for each scenario at 0.25° resolution (https://luh.umd.edu/). We converted to 0.05° resolution assuming the same land cover fractions for all 25 grid cells around the LUH2 grid cells. We considered the following land cover transitions: forest to managed grassland, forest to cropland, and natural grassland to cropland (and reverse transitions for future scenarios). Transitions in areas suitable for only two land cover types were also included. Conversions of natural grasslands to managed grasslands were assumed not to affect productivity. We assumed the original land cover of a grid cell to be either forest (i.e., potential forest cover > 36.3%) or natural grassland and accordingly multiplied the converted areas by the differences in potential GPP derived from our RF approach. Our broad forest definition including open tree cover (see above) and the fact that we assumed a change from 100 to 0% forest area in deforested grid cells results in a total historical deforestation area substantially larger than estimated in a recent study (2.4 Mkm^2^ vs. 1.6 Mkm^2^)^61^. These assumptions, however, do not impair our GPP estimate as our approach implicitly accounts for gradients in forest productivity (open forests tend to have lower GPP than closed forests). To test the sensitivity of the resulting GPP reduction we also applied the potential GPP maps from our uncertainty analysis to historical land-use changes (Supplementary Fig. S6). For future land cover changes we investigated changes over the 2015–2100 period for all available LUH2 scenarios: SSP1-1.9, SSP2-2.6, SSP4-3.4, SSP5-3.4, SSP2-4.5, SSP4-6.0, SSP3-7.0, and SSP5-8.5. Land-use activities in these scenarios range from large-scale deforestation (e.g., SSP3-7.0) to reforestation (e.g., SSP1-1.9) (Supplementary Fig. S7).

### Earth System Models

We compared the potential GPP estimated by our RF algorithm to simulations of eight ESMs participating in CMIP6 (CESM2-CLM5^62^, CNRM-ESM2.1-Surfex 8.0c^63^, EC-Earth3-Veg-LPJ-GUESSv4^64^, GFDL-ESM4-GFDL-LM4.1^65^, IPSL-CM6A-LR-ORCHIDEEv2.0^66^, MIROC-ES2L-MATSIRO6.0 + VISIT-e ver.1.0^67^, MPI-ESM1-2-LR-JSBACH3.20^68^, UKESM1-0-LL-JULES-ES-1.0^69^) with an explicit representation of natural vegetation and at least one agricultural land cover class (cropland or managed grassland) in their vegetation sub-model. We selected these ESMs so that all vegetation models implemented in more than one ESM were represented only once (e.g., the JSBACH vegetation model is a component of both MPI-ESM1-2-LR and AWI-ESM). For each ESM, the variable *gppLut* was downloaded from the CMIP6 archive (https://esgf-data.dkrz.de/search/cmip6-dkrz/) for the historical simulations. These files contain simulated GPP for natural vegetation, pasture, and cropland for which we calculated the 2001–2014 mean (2014 is the last year of the historical period). ESMs use fractional land covers for each grid cell, meaning that climatic drivers are inherently the same for all land cover types within a grid cell and simulated productivities can therefore be directly compared. As ESMs differ in their spatial resolution we bilinear remapped all output to 0.05° resolution using Climate Data Operators^33^ to allow for a fair comparison across models. To assess the sensitivity of our results to the interpolation method we also tested conservative remapping which results in slightly different maps (Supplementary Fig. S15) and usually larger model biases (Supplementary Table 2). In addition, ESMs differ in where they simulate forests in natural vegetation areas, and therefore we removed all grid cells from the comparison where at least one ESM simulated no tree productivity/cover/biomass in order to avoid comparing the GPP of natural grasslands to managed grasslands. We provide maps based on the original output for each ESM in Supplementary Fig. S10.

### FLUXNET data

We compared our predictions of potential GPP to FLUXNET Tier 1 eddy covariance measurements (Supplementary Fig. S16)^70^. We included all forest, woody savannah (classified as forest), grassland and cropland sites^21^ which were located in suitable areas for the respective land cover. Mean GPP was calculated as the mean of the GPP estimates based on the night-time (GPP_NT_VUT_REF) and day-time (GPP_DT_VUT_REF) partitioning method. As some sites only had a few years of data, all available years were considered (i.e., site mean GPP was calculated for a different time period than 2001–2015). Comparisons were made with the potential GPP in the respective grid cell in which the site was located (i.e., not calibrated to site conditions).

## Supplementary Information

Supplementary Information.

## Data Availability

All datasets used in this study are publicly available. The GOSIF dataset can be downloaded from http://data.globalecology.unh.edu/data/GOSIF_v2/. CMIP6 output can be downloaded from https://esgf-data.dkrz.de/search/cmip6-dkrz/ while the LUH2 scenarios are available from https://luh.umd.edu/. The potential GPP maps can be downloaded as text files from https://figshare.com/articles/dataset/Krause_et_al_2022_Scientific_Reports/21304755.
