# Supplementary material for: Quantifying the impacts of land cover change on gross primary productivity globally
Source: Sci Rep. 2022 Nov 1;12:18398. doi: 10.1038/s41598-022-23120-0 (PMC9626452; doi:10.1038/s41598-022-23120-0)
Supplement: Supplementary file 1 — Supplementary Information. [file 41598_2022_23120_MOESM1_ESM.docx]

Supplementary Information for

**Quantifying the impacts of land cover change on gross primary productivity globally**

Andreas Krause, Phillip Papastefanou, Konstantin Gregor, Lucia S. Layritz, Christian S. Zang, Allan Buras, Xing Li, Jingfeng Xiao, Anja Rammig

Supplementary Discussion 1: Maps of the most productive land cover derived from the uncertainty analysis

Our findings may be sensitive to the forest definition, different datasets of current land cover, potential forest cover, climate, and GPP, or alternative modelling techniques. However, our uncertainty analysis reveals that forests typically being the most productive land cover is confirmed for a range of alternative datasets and approaches (Supplementary Fig. S2). Replacing our potential forest map with potential forest cover from LUH2 yields a much smaller suitable area compared to our default approach (Supplementary Fig. S2a+b). However, the LUH2 forest map, which is based on simulated above-ground biomass from the MIAMI model, substantially underestimates potential forest cover in several regions (Supplementary Fig. S13). Using the MODIS land cover product instead of ECA-CCI land cover substantially decreases the area where grasslands (4%) or croplands (10%) are more productive than forests (Supplementary Fig. S2c). Replacing CHELSA climate predictor variables with WorldClim climate results in almost the same productivity patterns (Supplementary Fig. S2d). Similarly, an alternative RF algorithm in Python basically produces the same map as the default one (Supplementary Fig. S2e), while an alternative machine learning approach (Deep Neural Networks) increases the area where grasslands (16%) or croplands (25%) are the most productive land cover (Supplementary Fig. S2f). A much simpler nearest-neighbour approach, which for each grid cell assigns the respective GPP values of the nearest forest, grassland, and cropland grid cell, also slightly increases the area where grasslands and croplands are most productive (Supplementary Fig. S2g). Using four alternative GPP products results in relatively similar patterns of the most productive land cover as our default approach, even though some deviations can be found (Supplementary Fig. S2h+k). Finally, replacing the MODIS GPP product with MODIS NPP, the area where grasslands or croplands are the most productive land cover becomes substantially larger (Supplementary Fig. S2l). Even though at most locations (61% compared to 79% for MODIS GPP) forests are still more productive, this suggests a lower carbon use efficiency for forests than for agricultural land cover types. Overall, all approaches agree on forests as the most productive land cover type at most locations.

Supplementary Discussion 2: Evaluation metrics and spatial autocorrelation

The high R^2^ (forest: 0.99, grassland: 0.98, cropland: 0.97) and low RMSE (forest: 109 g C m^-2^ yr^-1^, grassland: 87 g C m^-2^ yr^-1^, cropland: 114 g C m^-2^ yr^-1^) suggest a very good prediction skill of our RF algorithm. For comparison, the RF studies mentioned in the methods section reported R^2^ values ranging between 0.28 and 0.96 ^1-5^. Potential GPP also shows reasonable agreement with FLUXNET sites: while there is little prediction skill for croplands, forest and especially grassland sites have strong correlations between potential and tower GPP (Supplementary Fig. S15), taking into account that environmental conditions in our model were not calibrated to site conditions and that the compared time periods are different. The favourable statistical measures in our study can partly be explained by the very large training data (> 500,000 grid cells per land cover type) and the fact that we conduct a global analysis involving very different climatic conditions. In fact, restricting the investigated area to the tropics (22.24°S-22.24°N) decreases forest R^2^ to 0.96 and increases RMSE to 145 g C m^-2^ yr^-1^. Reducing the forest training data to only 1% of all global observations (still >20,000 randomly selected grid cells) decreases forest R^2^ to 0.97 and increases RMSE to 187 g C m^-2^ yr^-1^. Related to that, it is known that spatial autocorrelation can explain much of the prediction skill of RF algorithms (i.e., simply using coordinates as predictors can result in comparable evaluation statistics compared to predictions based on environmental variables) ^6,7^. Using the same approach (i.e., coordinates as sole predictors) in our model somewhat reduces R^2^ and increases RMSE (forest R^2^: 0.98, RMSE: 129 g C m^-2^ yr^-1^; grassland R^2^: 0.98, RMSE: 97 g C m^-2^ yr^-1^; cropland R^2^: 0.97, RMSE: 129 g C m^-2^ yr^-1^), meaning that even though spatial autocorrelation exists in our data, including environmental input variables improves evaluation metrics, especially RMSE. We additionally test the applicability of our approach by performing leave-one-out cross validation including spatial buffers around the test observations ^7^. As expected this leads to decreasing model performance, but we still find a good prediction skill for locations hundreds of kilometres away from the next training data (Supplementary Fig. S3). As almost all grid cells are located within 400 (forest: 99.7%; grassland: 94.2%; cropland: 97.4%), often even within 100 kilometres (forest: 94.4%; grassland: 61.2%; cropland: 83.7%) from the next sample grid cell (Supplementary Fig. S4), we can assume a high accuracy in almost all locations (even though typically lower than reported for the testing data). The RF model based on environmental predictors is also superior compared to a model without any predictors and, more importantly, the coordinate-only model. Furthermore, we repeat our original analysis using grid cells from Eurasia, Africa, and Australia as training data and those from America as evaluation data. Results again confirm a reasonable prediction skill in regions far away from any training data even though evaluation metrics naturally decline (forest R^2^: 0.92, RMSE: 355 g C m^-2^ yr^-1^; grassland R^2^: 0.80, RMSE: 382 g C m^-2^ yr^-1^; cropland R^2^: 0.81, RMSE: 302 g C m^-2^ yr^-1^). In this case, the coordinate-only model performs much worse (forest R^2^: 0.27, RMSE: 1071 g C m^-2^ yr^-1^; grassland R^2^: 0.09, RMSE: 743 g C m^-2^ yr^-1^; cropland R^2^: 0.00, RMSE: 733 g C m^-2^ yr^-1^) than the full model, emphasizing the importance of environmental variables for predictions in regions far away from any training data.

Supplementary Discussion 3: Random forests variable importance

Annual mean temperature, minimum temperature of the coldest month, and annual temperature range are the most important predictor variables for forest GPP (Supplementary Fig. S12). Mean annual precipitation and precipitation of the driest month are the most important predictors for grassland GPP. Cropland GPP can mainly be explained by annual temperature range, annual mean precipitation, and relative humidity. Note that RF variable importance may be affected by variable collinearity (e.g., between minimum monthly temperature and mean annual temperature, see Supplementary Fig. S17) but this generally does not impair the model’s prediction skill.

**Supplementary Tables and Figures**

Supplement Table 1: Average potential GPPs in different continents and major countries. Note that only grid cells suitable for all three land cover types were included.

| **Continent/Country** | **Potential forest GPP [g C m^-2^ yr^-1^]** | **Potential grassland GPP [g C m^-2^ yr^-1^]** | **Potential cropland GPP [g C m^-2^ yr^-1^]** |
| --- | --- | --- | --- |
| Africa | 2057 | 1542 | 1961 |
| Asia (excluding Russia) | 1932 | 1296 | 1651 |
| Europe (excluding Russia) | 1253 | 1265 | 1173 |
| North America | 1352 | 1287 | 1290 |
| South and Central America | 2658 | 2025 | 2359 |
| Russia | 1110 | 1039 | 906 |
| China | 1524 | 1109 | 1278 |
| Canada | 1040 | 1048 | 1124 |
| US | 1464 | 1373 | 1349 |
| Brazil | 2799 | 2250 | 2425 |
| Australia | 1132 | 1062 | 1379 |
| India | 1535 | 979 | 1270 |
| Argentina | 1593 | 1454 | 1498 |
| Democratic Republic of the Congo | 2573 | 1945 | 2656 |

Supplement Table 2: Grid cell-level agreement in GPP between ESM simulations and our RF prediction. Numbers outside of the brackets are for bilinear remapping of ESM output to 0.05° spatial resolution, numbers in brackets are for conservative remapping. Note that some of the differences between both options can be explained by conservative mapping resulting in ~27% more grid cells for this comparison.

| **ESM** | **Forest** | | **Grassland** | | **Cropland** | |
| --- | --- | --- | --- | --- | --- | --- |
|  | **R^2^** | **RMSE** | **R^2^** | **RMSE** | **R^2^** | **RMSE** |
| ESM ensemble  mean | 0.63 (0.60) | 491 (520) | 0.43 (0.37) | 479 (511) | 0.69 (0.66) | 383 (419) |
| CESM-CLM | 0.48 (0.46) | 731 (785) | 0.00 (0.00) | 820 (880) | - | - |
| CNRM-SURFEX | 0.42 (0.44) | 880 (878) | - | - | 0.17 (0.20) | 682 (708) |
| EC-EARTH-LPJ-GUESS | 0.18 (0.11) | 785 (899) | 0.02 (0.04) | 1027 (1063) | 0.23 (0.16) | 638 (729) |
| GFDL-LM | 0.47 (0.43) | 641 (700) | 0.24 (0.19) | 953 (1017) | 0.43 (0.42) | 1127 (1194) |
| IPSL-ORCHIDEE | 0.62 (0.58) | 732 (774) | - | - | 0.55 (0.44) | 463 (549) |
| MIROC-VISIT | 0.28 (0.17) | 685 (813) | 0.09 (0.03) | 662 (747) | 0.31 (0.21) | 613 (756) |
| MPI-JSBACH | 0.34 (0.30) | 701 (788) | 0.14 (0.09) | 673 (737) | 0.47 (0.45) | 1280 (1332) |
| UKESM-JULES | 0.55 (0.49) | 565 (638) | 0.43 (0.38) | 1040 (1172) | 0.65 (0.60) | 838 (938) |

Supplement Table 3: Datasets used in this study.

| Abbreviation | Full name | Spatial resolution | Temporal resolution | Available time period | Website | Reference |
| --- | --- | --- | --- | --- | --- | --- |
| GOSIF GPP | Global, OCO-2 based SIF product gross primary production | 0.05° | 8-day or monthly | 2000 -2021 | https://globalecology.unh.edu/data/GOSIF-GPP.html | ^8^ |
| VPM GPP | Vegetation  photosynthesis model gross primary productivity | 500 m, 0.05° or 0.5° | 8-day, monthly or yearly | 2000 -2016 | https://figshare.com/collections/A_global_moderate_resolution_dataset_of_gross_primary_production_of_vegetation_for_2000-2016/3789814 | ^9^ |
| Yebra et al. GPP | - | 0.05 | monthly | 2001 -2012 | http://wald.anu.edu.au/data_services/data/global-0-05-gross-primary-production-estimates/ | ^10^ |
| FLUXCOM RS | FLUXCOM remote sensing | 30 arc sec | 8-day | 2001 -2015 | https://www.fluxcom.org/ | ^11,12^ |
| MODIS MOD17A3 GPP | - | 30 arc sec | yearly | 2000 - 2013 | http://www.ntsg.umt.edu/project/mod17 | ^13^ |
| MODIS MOD17A3 NPP | - | 30 arc sec | yearly | 2000 - 2013 | http://www.ntsg.umt.edu/project/mod17 | ^13^ |
| FLUXNET | Flux networks | sites | half-hourly or yearly | site-specific | https://fluxnet.org/ | ^14^ |
| ESA-CCI | European Space Agency Climate Change Initiative land cover | 300 m | yearly | 1992 -2020 | https://cds.climate.copernicus.eu/ | ^15^ |
| MODIS MCD12C1 | Moderate Resolution Imaging Spectroradiometer (MODIS) Land Cover Climate Modeling Grid | 0.05° | yearly | 2001 -2020 | https://lpdaac.usgs.gov/products/mcd12c1v006/ | ^16^ |
| Potential natural vegetation map | - | 30 arc sec | - | presence | https://dataverse.harvard.edu/dataset.xhtml?persistentId=doi:10.7910/DVN/QQHCIK | ^5^ |
| CHELSA | Climatologies at high resolution for the earth’s land surface areas | 30 arc sec | monthly or climatologies | 1979 -2018 or 1979 -2013 | https://chelsa-climate.org/ | ^17,18^ |
| WorldClim | - | 30 arc sec | climatologies | 1970 - 2000 | https://www.worldclim.org/data/worldclim21.html | ^19^ |
| Regridded Harmonized World Soil Database | - | 0.05° | - | presence | https://daac.ornl.gov/SOILS/guides/HWSD.html | ^20^ |
| ISIMIP2b nitrogen deposition | Inter-Sectoral Impact Model Intercomparison Project nitrogen deposition | 0.5° | monthly or yearly | 1850 - 2099 | https://www.isimip.org/gettingstarted/details/24/ | ^21^ |
| FAO nitrogen fertilization  / pesticides | Food and Agricultural Organization nitrogen fertilization  / pesticides | country-specific | yearly | 1990 -2017 | https://ourworldindata.org/grapher/nitrogen-fertilizer-application-per-hectare-of-cropland https://ourworldindata.org/grapher/pesticide-use-per-hectare-of-cropland |  |
| Gross Domestic Product map | - | 5 arc min | yearly | 1990 -2015 | https://datadryad.org/stash/dataset/doi:10.5061/dryad.dk1j0 | ^22^ |
| LUH2 | Land-Use Harmonization project | 0.25° | yearly | 850 -2100 | https://luh.umd.edu/ | ^23^ |
| CMIP6 ESMs | Coupled Model Intercomparison Project phase 6 Earth System Models | model-specific | monthly | 1850 -2014 (historical) | https://esgf-data.dkrz.de/search/cmip6-dkrz/ | ^24^ |


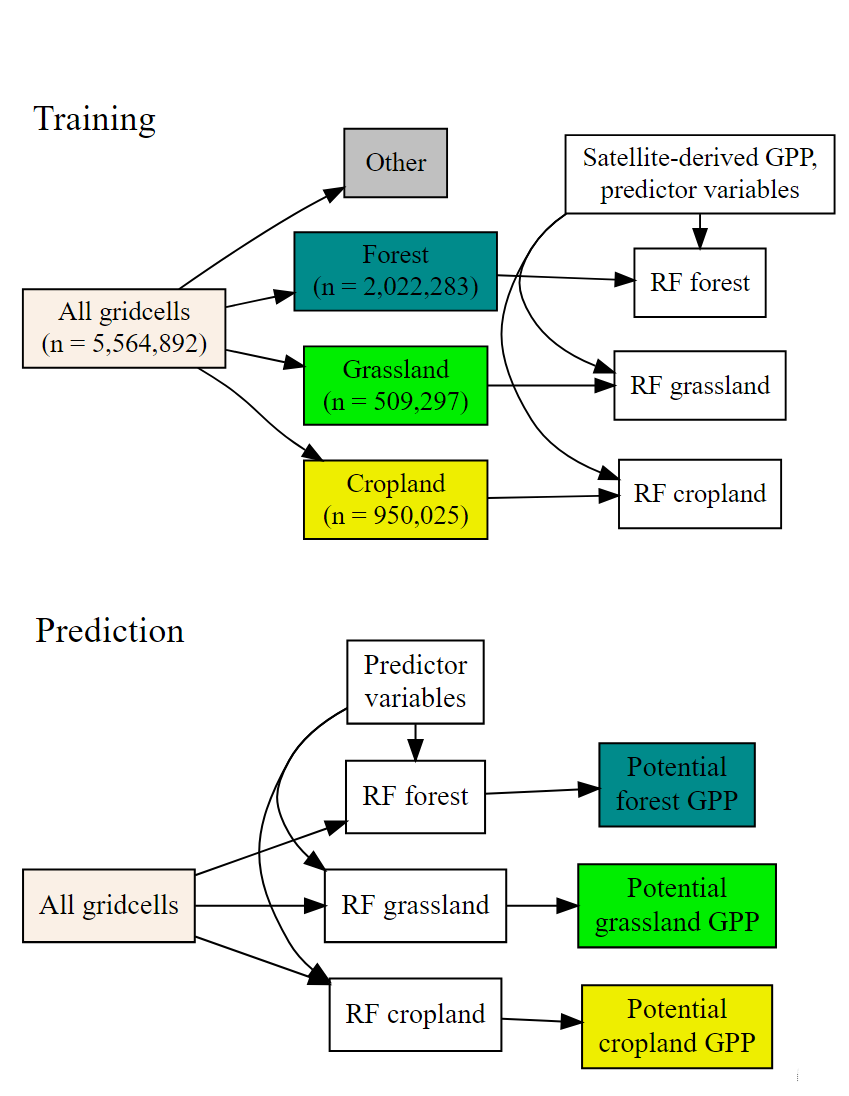


Fig. S1: Flowchart of our RF approach. The figure was created using R version 4.1.0 (<https://cran.r-project.org/>) ^25^.


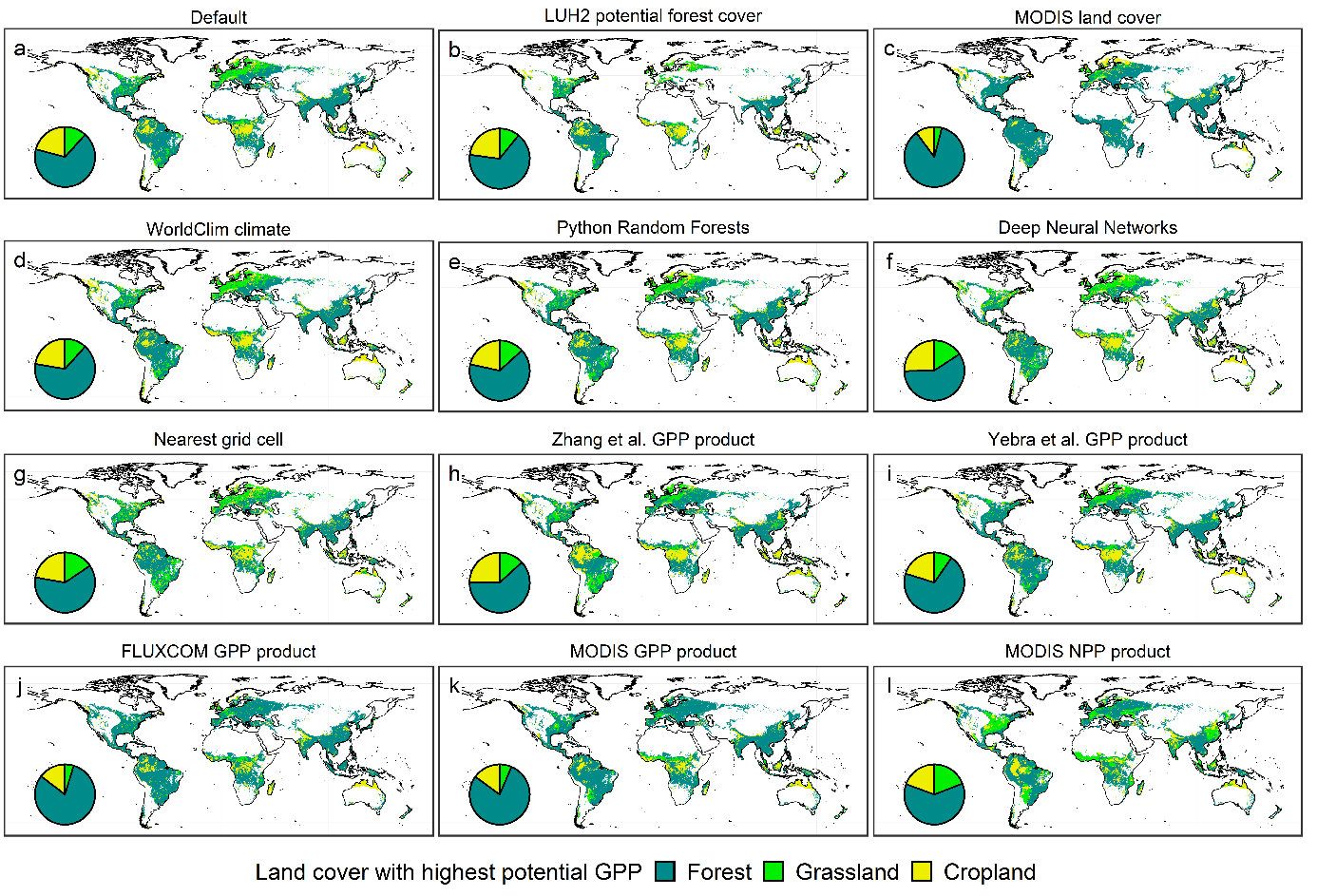


Fig. S2: Uncertainty analysis of our approach to different datasets and algorithms. Forests usually being the most productive land cover as seen in our default RF prediction (a) is confirmed using LUH2 potential forest cover (b), MODIS instead of ESA-CCI land cover (c), WorldClim instead of CHELSA climate (d), the *Python scikit-learn* RF algorithm instead of *R ranger* (e), an alternative machine-learning approach, Deep Neural Networks (f), a simple approach which just assigns the GPP value of the nearest forest, grassland, and cropland grid cell to each grid cell (g), and four alternative GPP products (h-k). In contrast to all other maps, l) is based on NPP (from MODIS) instead of GPP. For details about datasets and algorithms see Methods. Maps were created using R version 4.1.0 (<https://cran.r-project.org/>) ^25^.


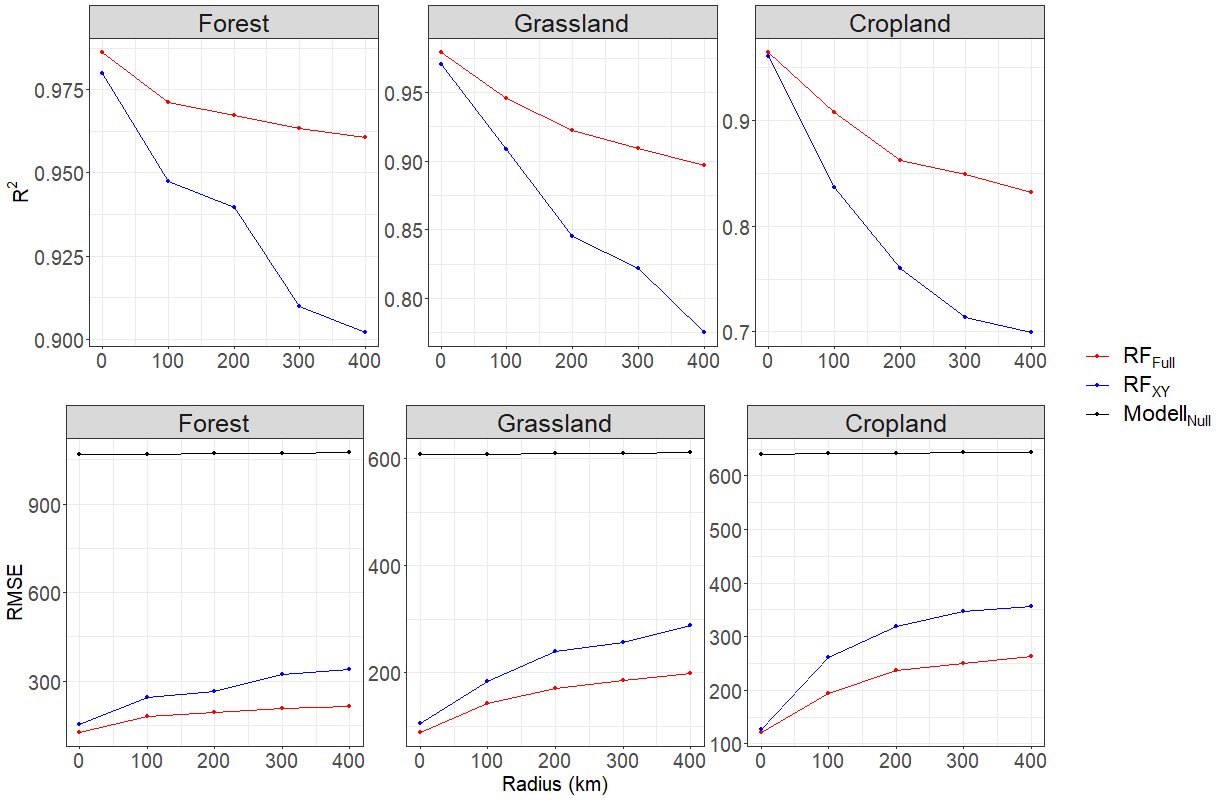


Fig. S3: Influence of data spatial structure on RF performance in terms of coefficient of determination (top) and root mean square error (bottom) following ref ^7^. Performance metrics are computed based on predicted *vs*. observed grid cell GPP as buffer radii for neighbouring grid cell exclusion increase in the buffered leave-one-out cross-validation. Validation data comprises 400 randomly selected grid cells for each land cover type. Predictions are made with the model based on 20 environmental predictor variables (RF_Full_) and the model based only on the grid cells’ geographic coordinates (RF_XY_). In addition to RF_Full_ and RF_XY_, the RMSE of a null model that systematically predicts the mean of the training data is plotted (Modell_NULL_). Due to the computational demand we reduced the number of decision trees to 100 and only used 25 and 54% of the training data for forest and cropland, respectively. The figure was created using R version 4.1.0 (<https://cran.r-project.org/>) ^25^.


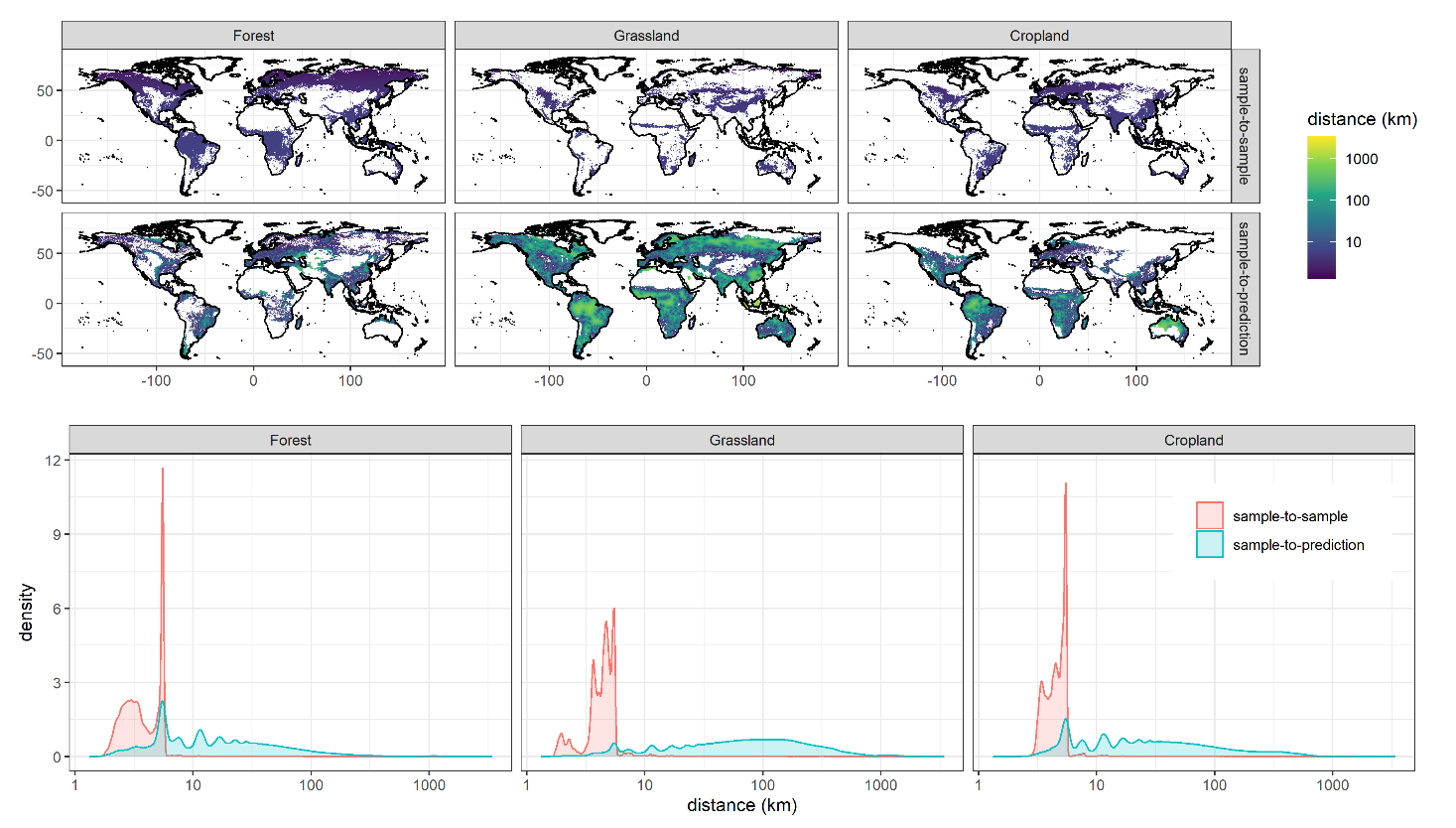


Fig. S4: Distribution of nearest neighbour distances. Sample-to-sample distances correspond to the training/testing dataset while sample-to-prediction distances correspond to grid-cells in which the respective land cover presently does not occur (but theoretically could). The density plot follows Fig. 1 from ref ^26^. The figure was created using R version 4.1.0 (<https://cran.r-project.org/>) ^25^.


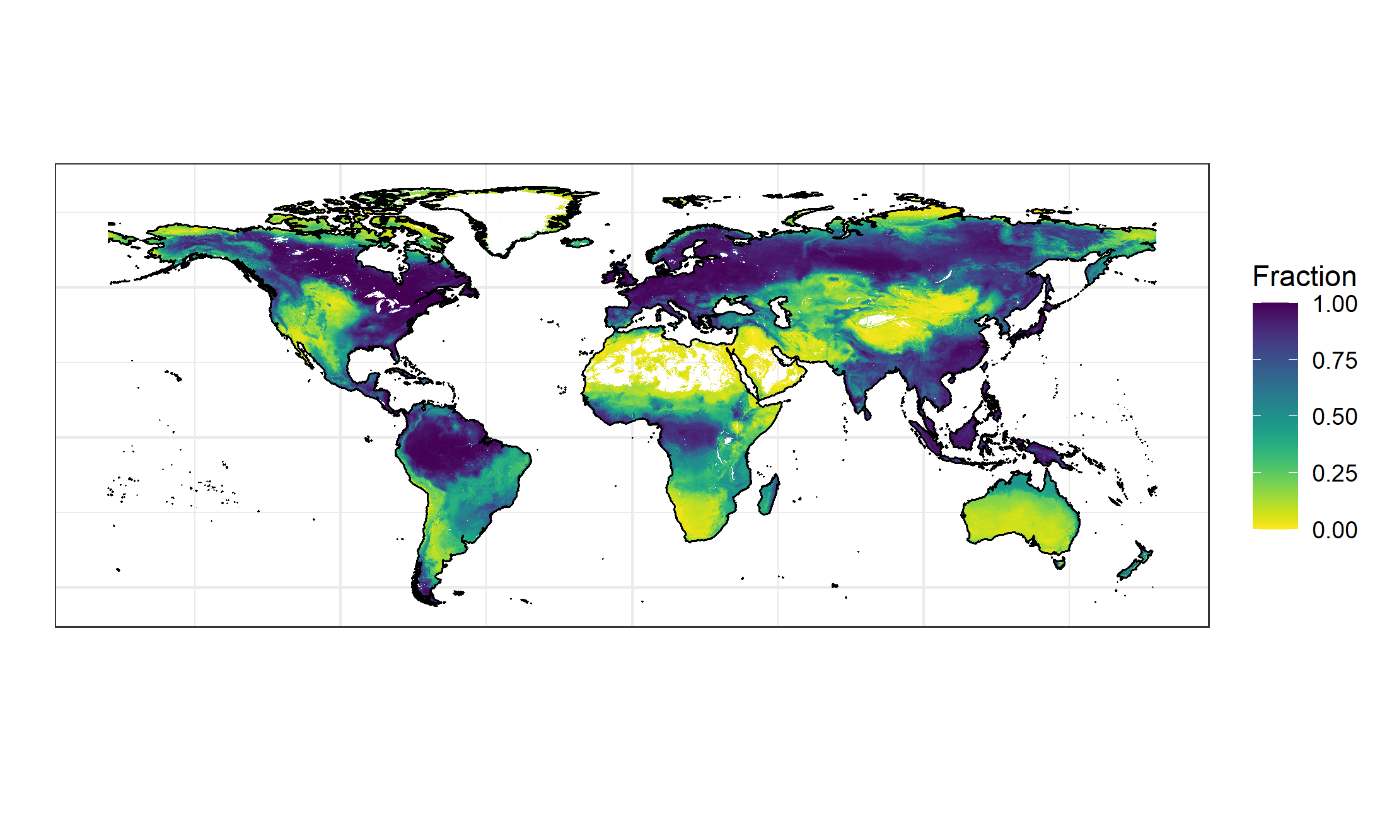


Fig. S5: Potential natural forest cover based on ref ^5^. We here assume a forest cover of 100% for the ten forest biomes in ref ^5^ and a forest cover of 30% for tropical savannahs and remapped to 0.05° spatial resolution. We then assume agricultural expansion according to LUH2 occurred at the expense of forests if the grid cell’s potential forest cover was > 36.3%. This threshold represents the 5^th^ percentile in our forest training data (i.e., currently forested grid cells). The map was created using R version 4.1.0 (<https://cran.r-project.org/>) ^25^.


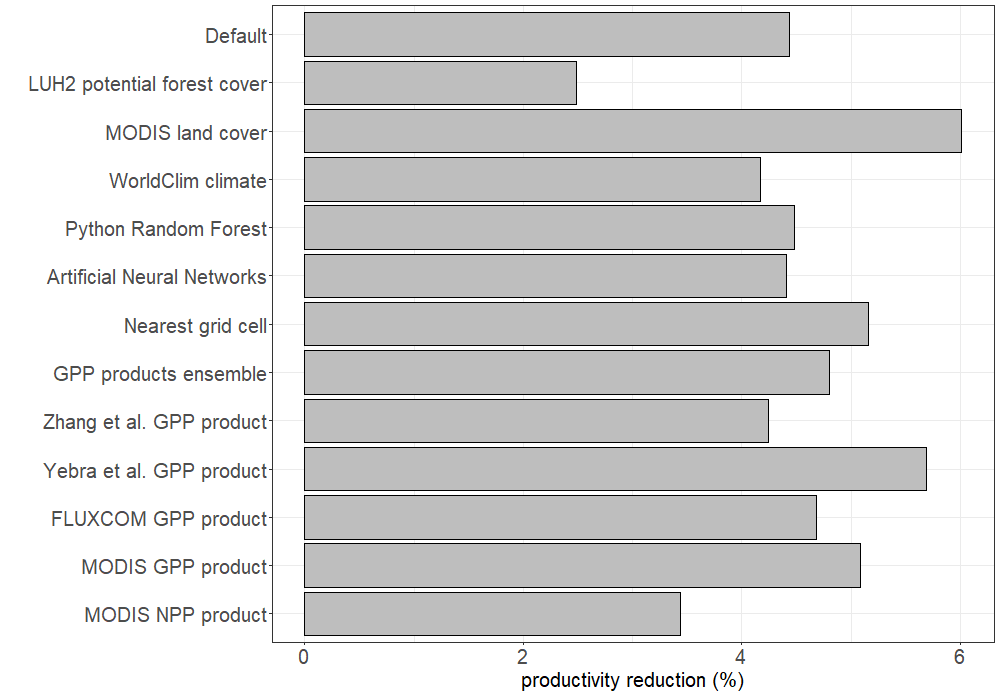


Fig. S6: Uncertainty analysis of the productivity reduction from historical agricultural expansion. For this, each alternative estimate of potential GPP (see Fig. S2) was combined with the LUH2 land-use reconstruction. The figure was created using R version 4.1.0 (<https://cran.r-project.org/>) ^25^.


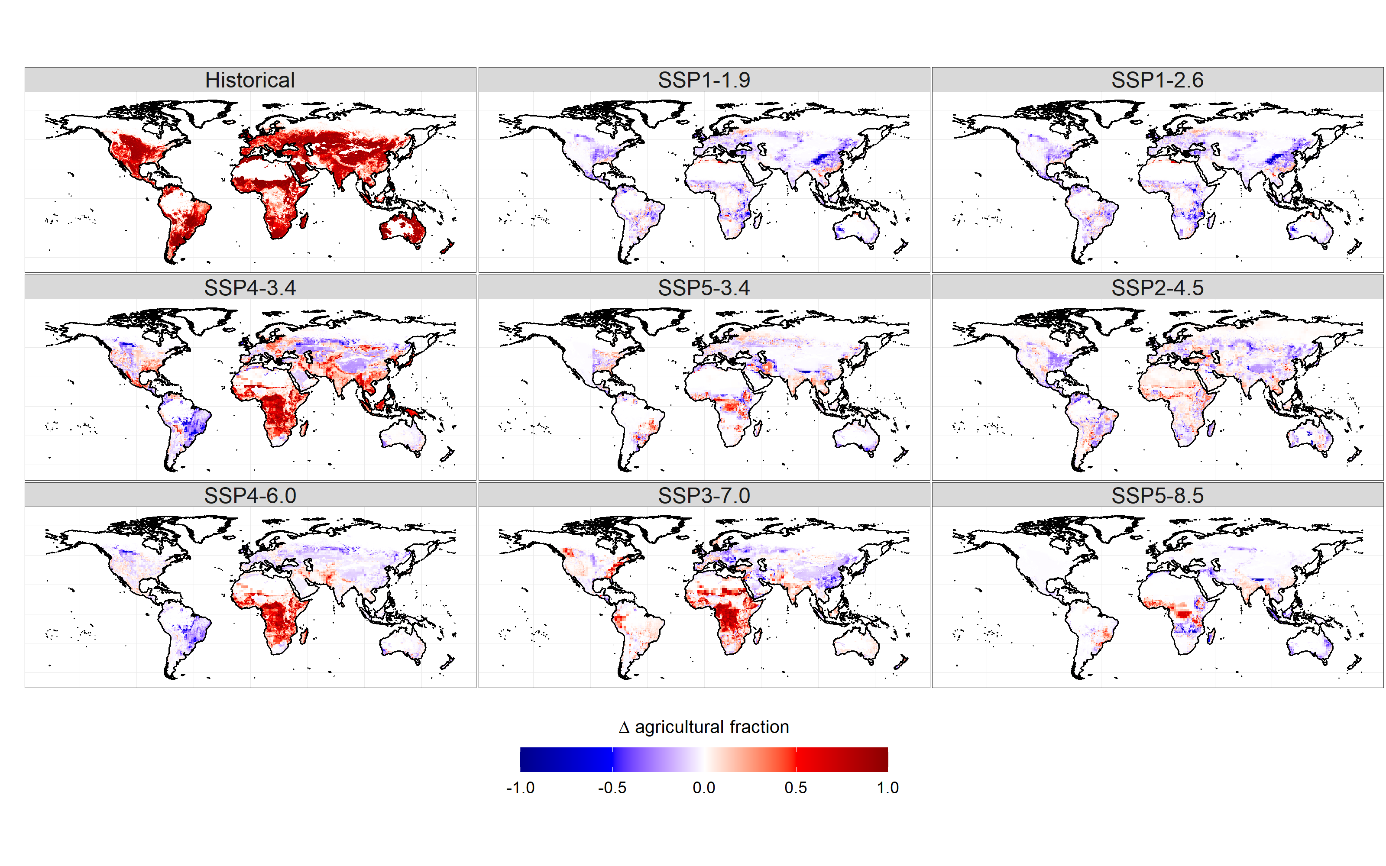


Fig. S7: Land-use changes in the LUH2 scenarios. Agriculture is the sum of croplands, pastures, and rangelands. Historical includes all agricultural expansion until year 2015, while the scenario maps show additional changes until year 2100. A value of 1 means the grid cell gets completely converted to cropland and/or managed grassland while negative values imply agricultural abandonment. Whether agricultural expansion occurs at the expense of forests or natural grasslands depends on the underlying forest map. Maps were created using R version 4.1.0 (<https://cran.r-project.org/>) ^25^.


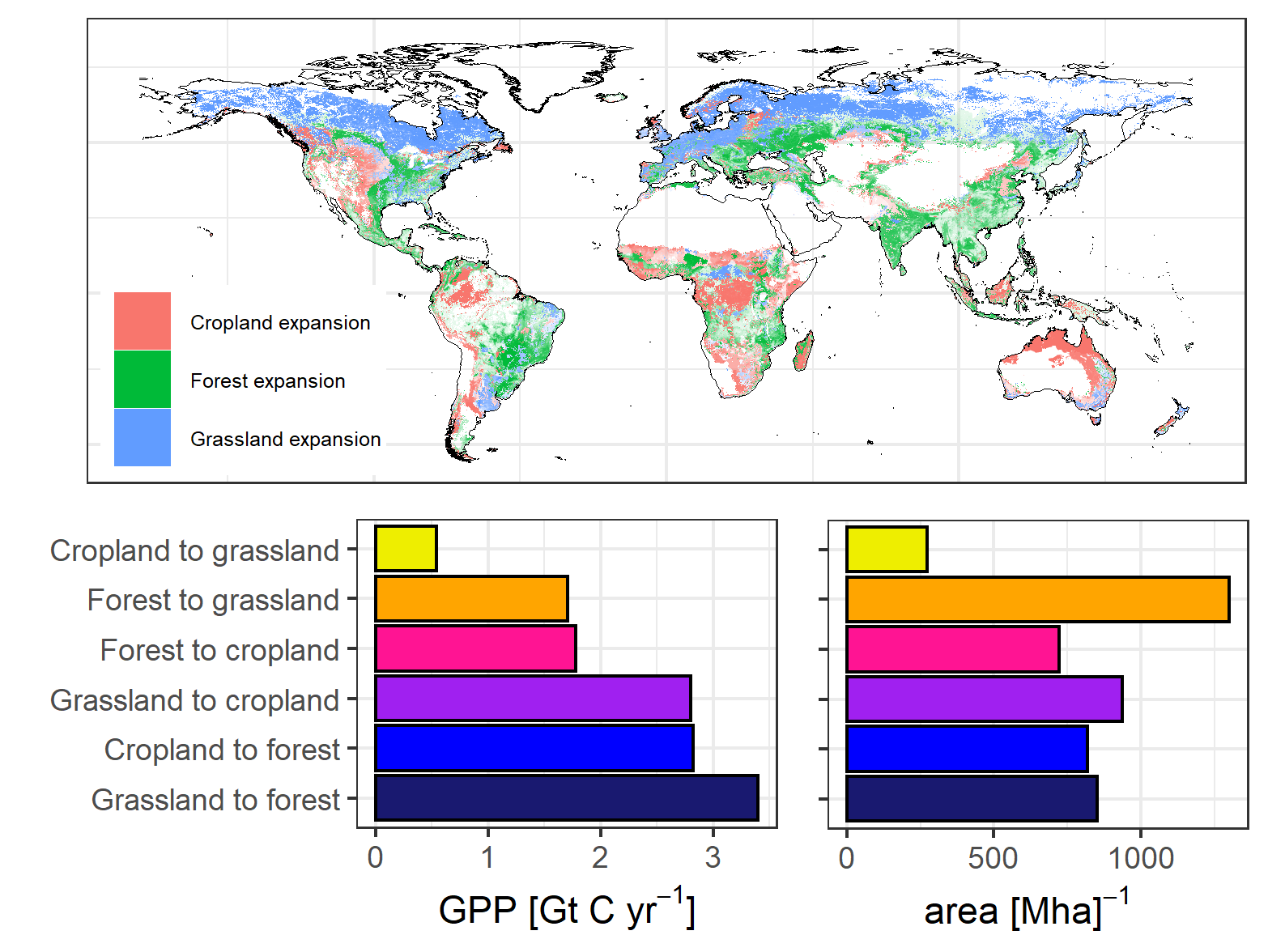


Fig. S8: Optimal land cover in terms of potential GPP (top) and global converted area and associated GPP increases (bottom). The colour shading in the map indicates the fraction of land that is presently not covered by the most productive land cover type. The figure was created using R version 4.1.0 (<https://cran.r-project.org/>) ^25^.


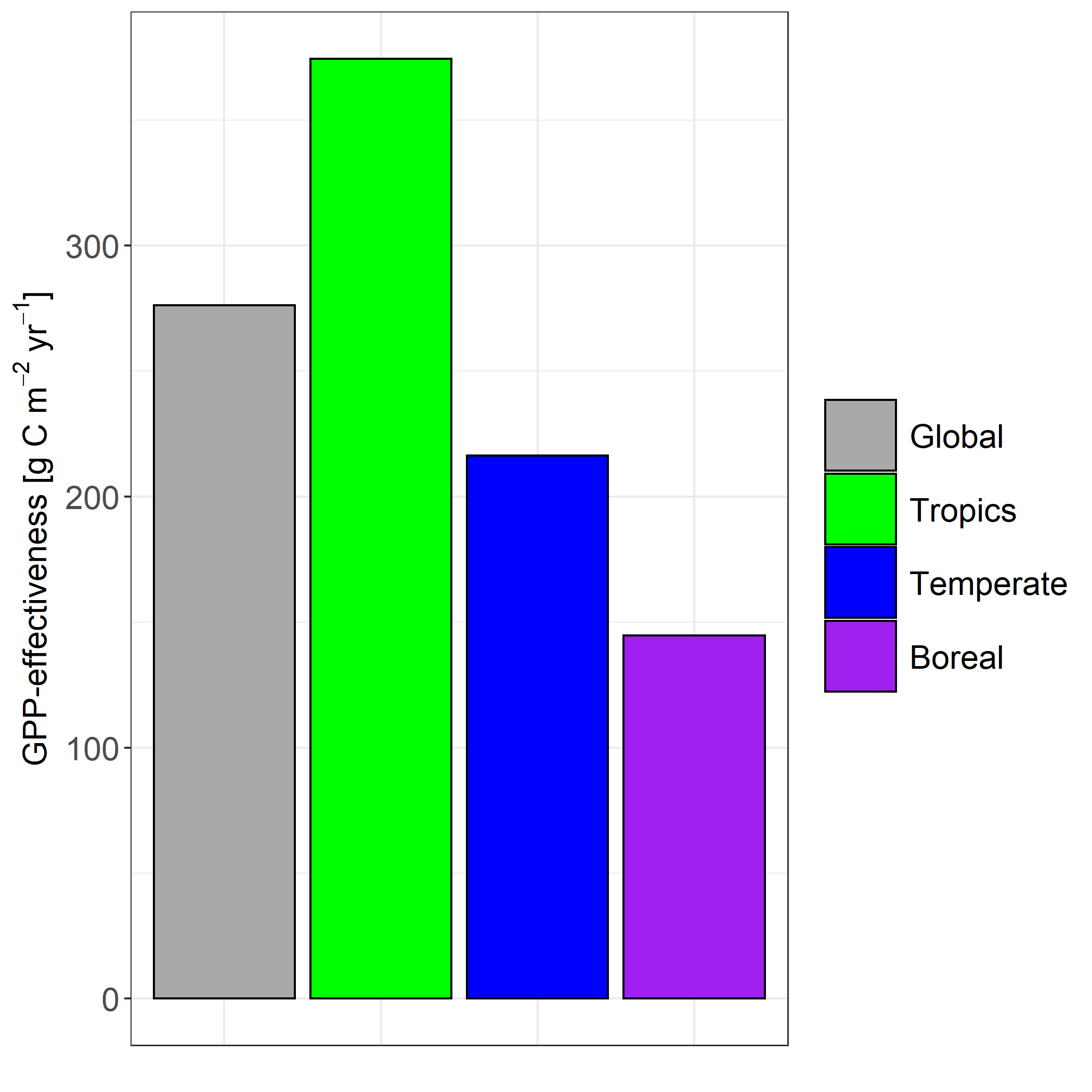


Fig. S9: Mean GPP-effectiveness (GPP increase per area) of agriculture-to-forest transitions in the tropics (22.24 °S–22.24°N), temperate (22.24°–48.23°), and boreal (>48.23°) regions. GPP-effectiveness was computed on present-day LUH2 cropland and managed grassland area, i.e., land presently not covered by forests. The figure was created using R version 4.1.0 (<https://cran.r-project.org/>) ^25^.


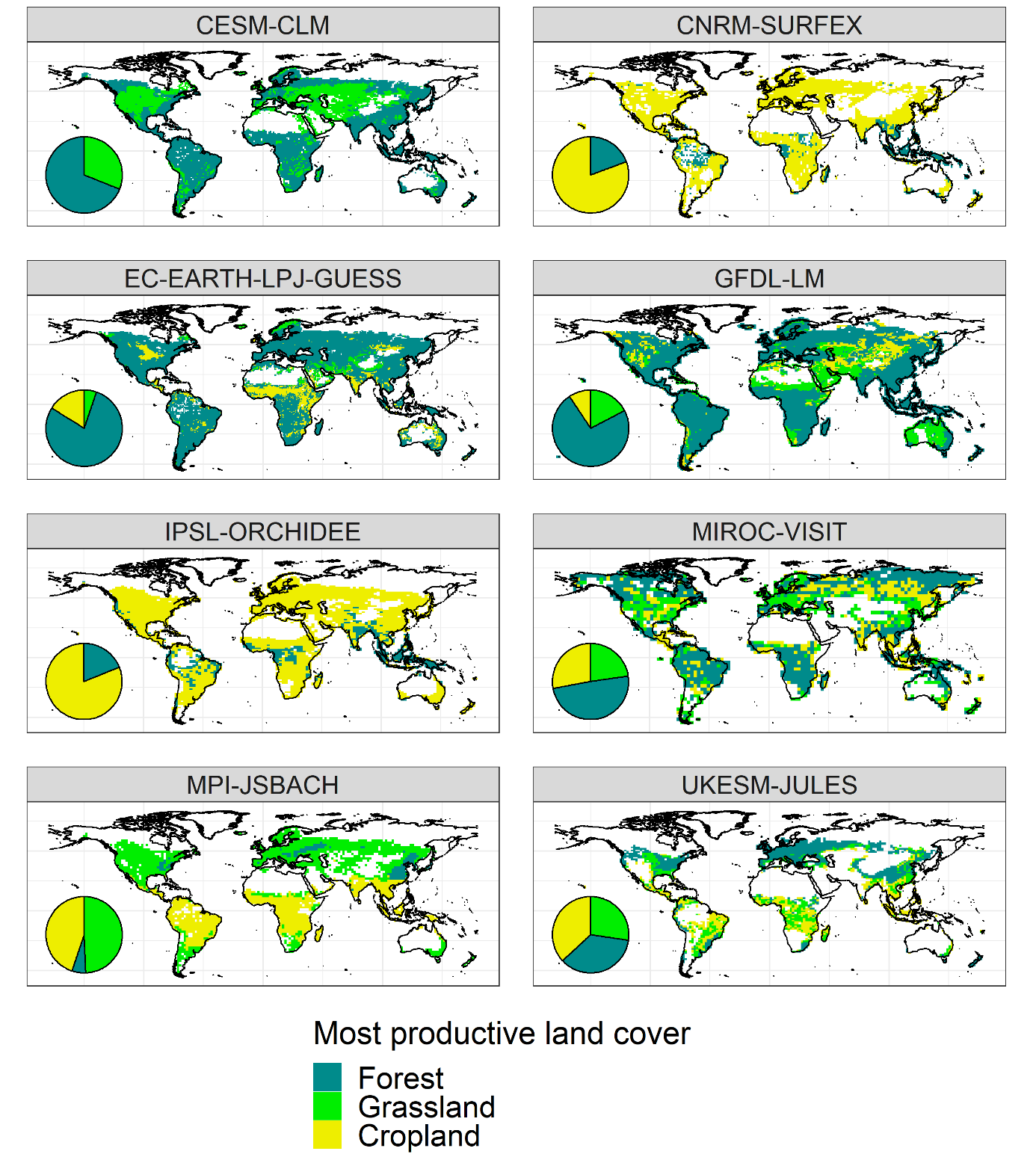


Fig. S10: Maps of the most productive land cover type for different CMIP6 ESMs, averaged over the 2001-2014 period. Pie charts give area-weighted totals. Note that forests here include all regions under natural vegetation where tree productivity/cover/biomass in the particular ESM is >0. Maps were created using R version 4.1.0 (<https://cran.r-project.org/>) ^25^.


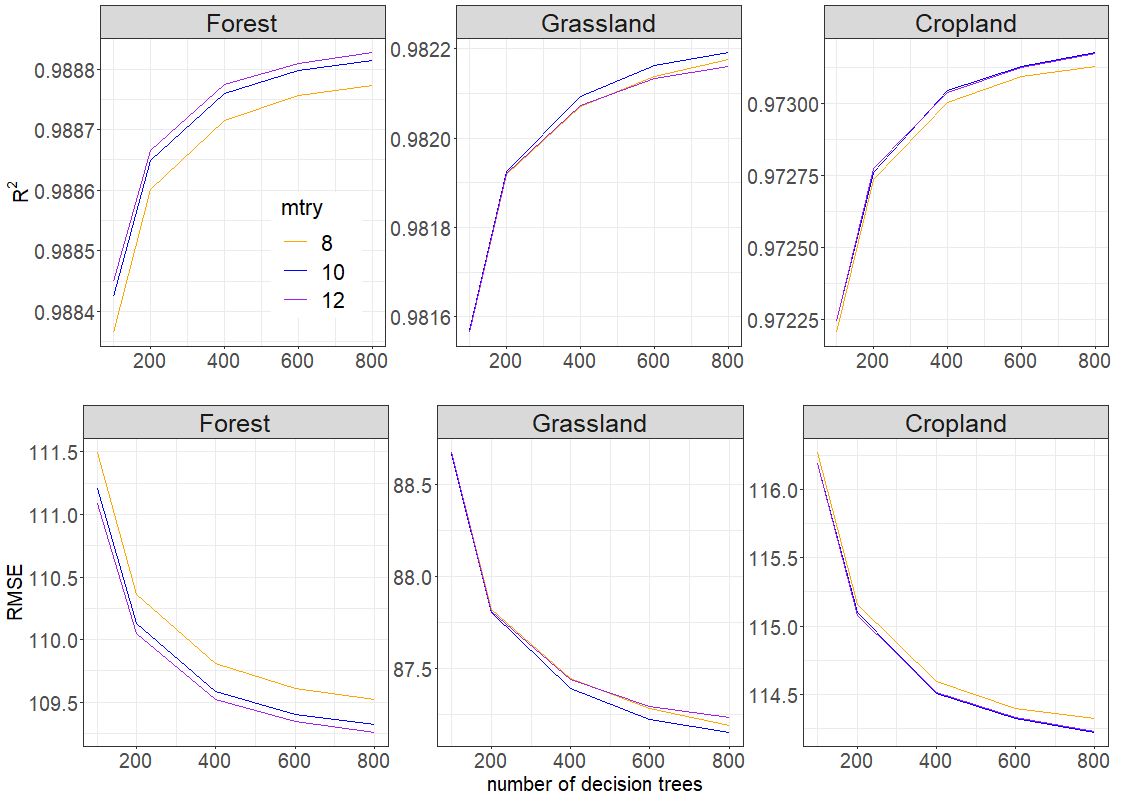


Fig. S11: RF performance in terms of coefficient of determination (top) and root mean square error (bottom) depending on varying number of variables to split at in each node (mtry) and number of individual decision trees. Performance metrics are computed on the out-of-bag (OOB) data, which serve as an internal testing dataset in the RF algorithm. In the final RF model we set mtry to 10 and the number of trees to 800. The figure was created using R version 4.1.0 (<https://cran.r-project.org/>) ^25^.


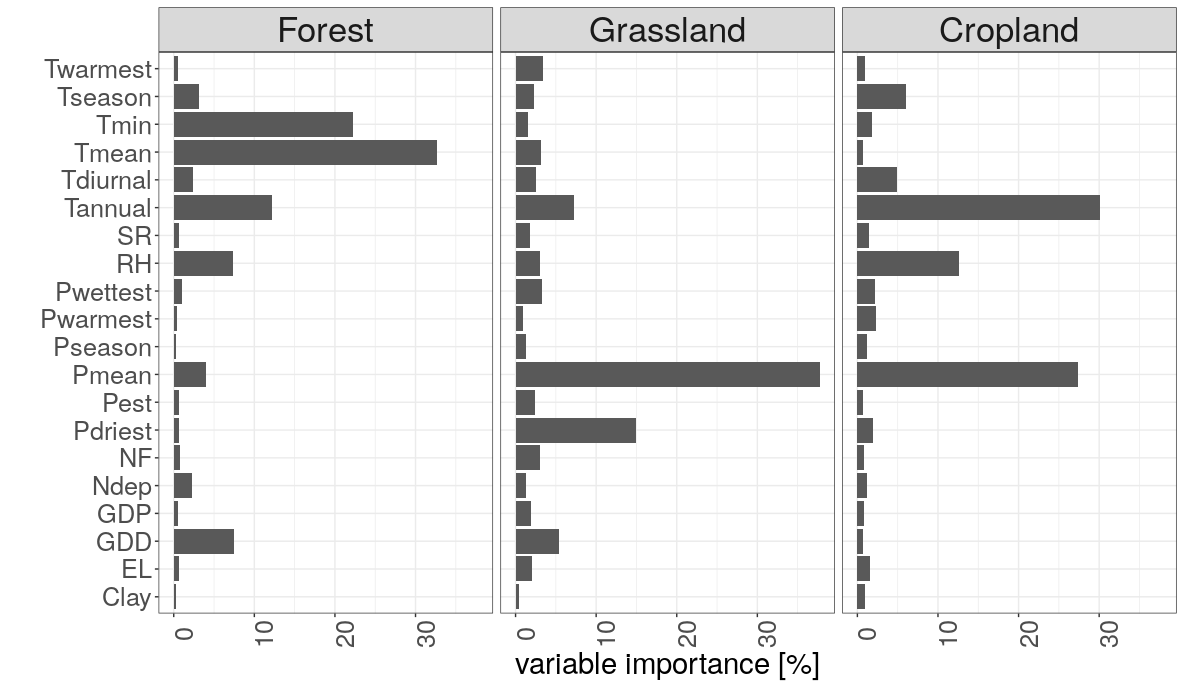


Fig. S12: Independent variable importance on the RF prediction skill in terms of impurity. Higher percentages imply a greater importance of the variable to accurately predict GPP. Note that variable importance can be impaired by collinearity between variables. The figure was created using R version 4.1.0 (<https://cran.r-project.org/>) ^25^.


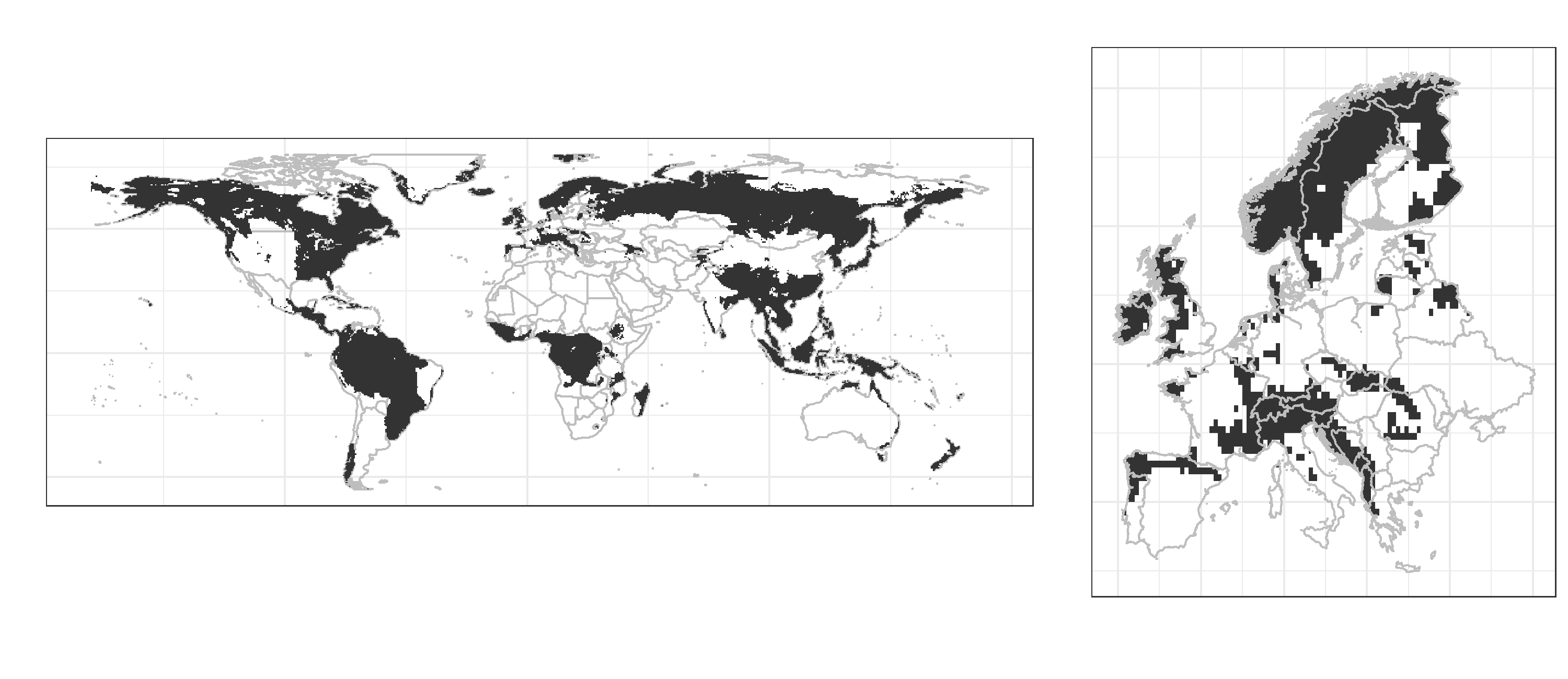


Fig. S13: Potential forest cover according to LUH2. In this product, grid cells with an above-ground biomass > 2 kg C m^-2^ according to the MIAMI model are classified as potentially forested. The LUH2 forest map clearly underestimates potential forest cover in several regions globally, including Central Europe, while e.g., overestimating over the Tibet plateau. Maps were created using R version 4.1.0 (<https://cran.r-project.org/>) ^25^.


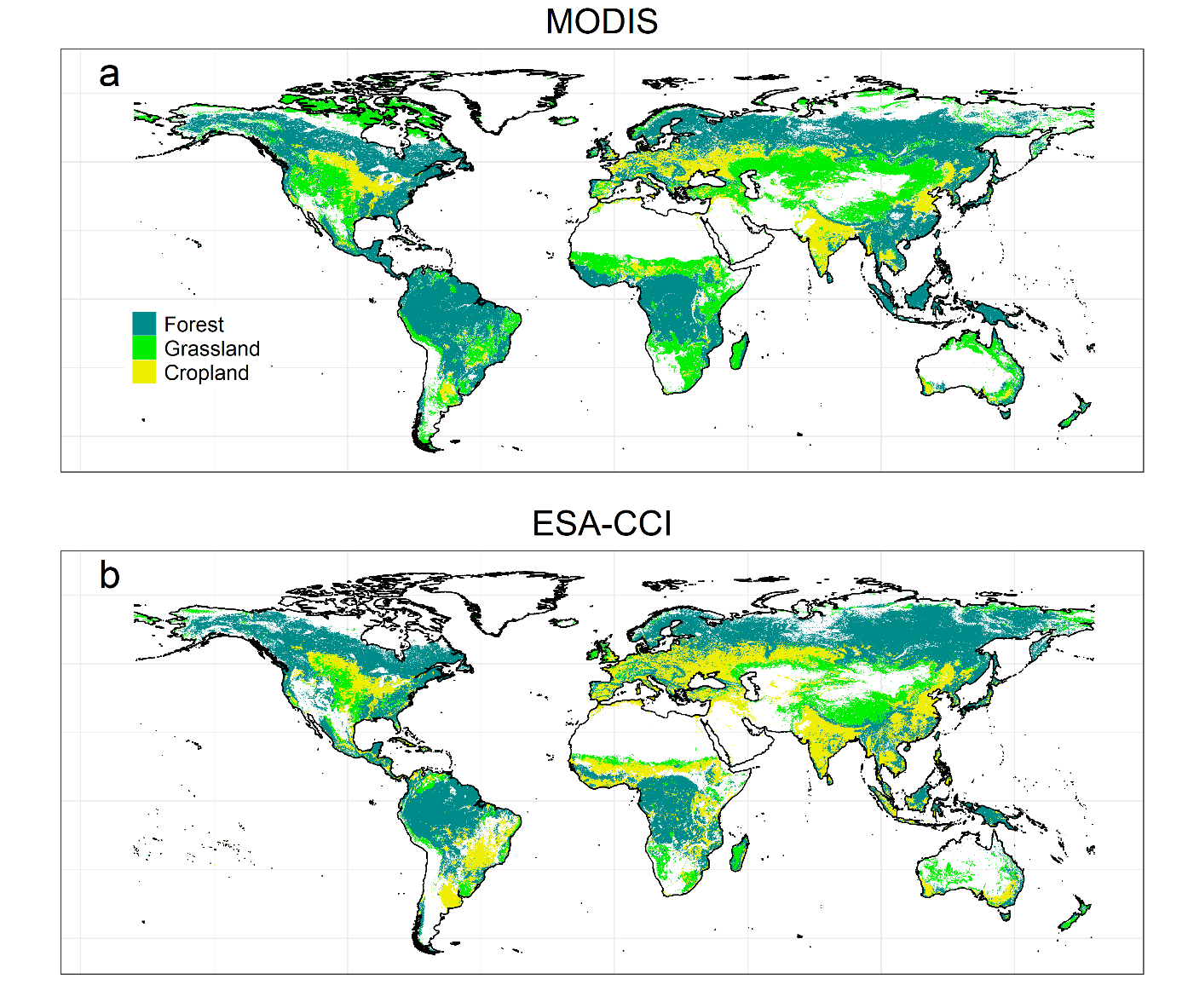


Fig. S14: Land cover maps according to the MODIS (a; used for Supplementary Fig. S2c) and ESA-CCI (b; our default product) land cover products. Only permanent forests, grasslands, and croplands (i.e., the training data) are shown. Maps were created using R version 4.1.0 (<https://cran.r-project.org/>) ^25^.


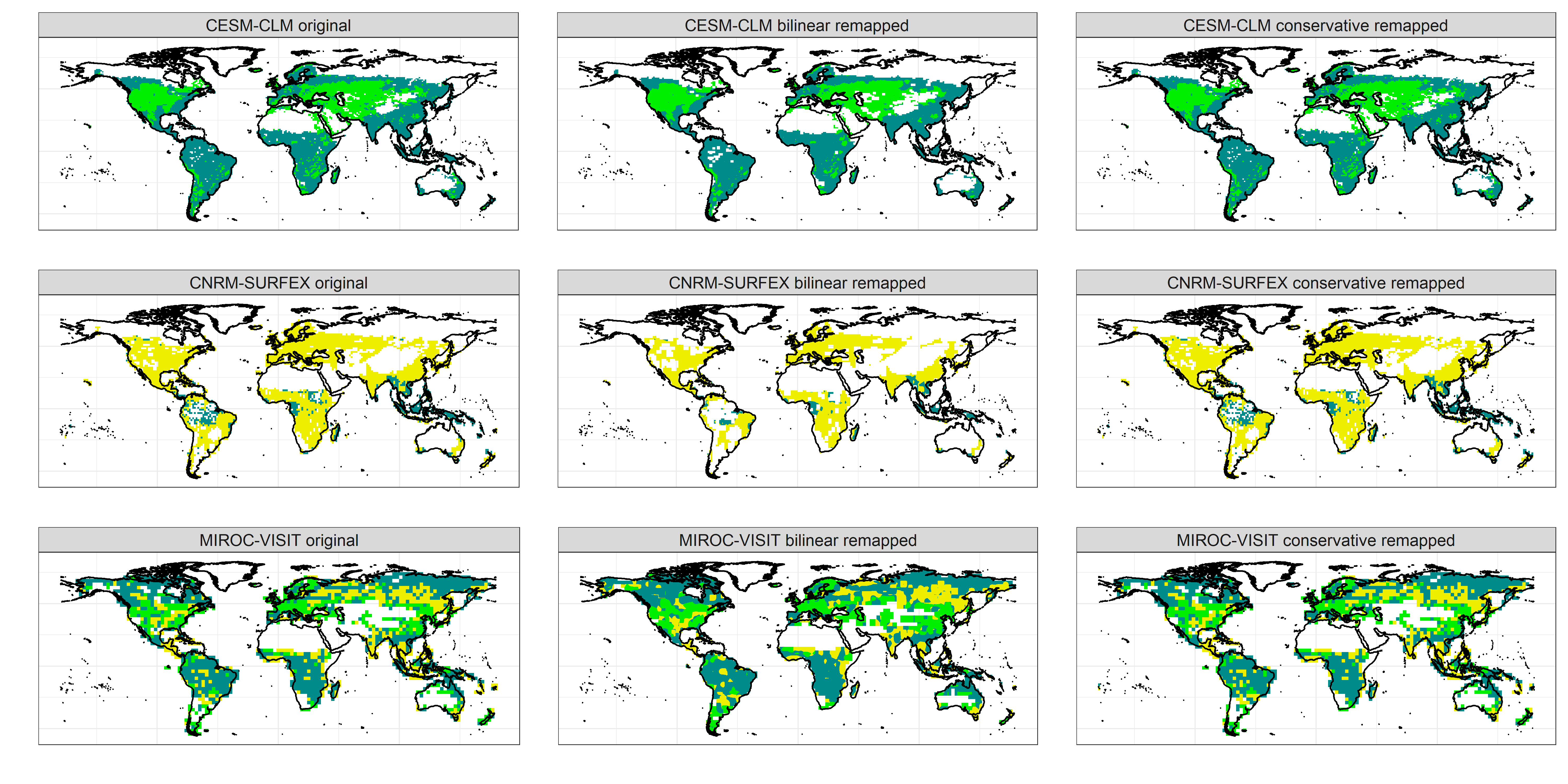


Fig. S15: Comparison of the most productive land cover type between outputs on the original spatial resolution of three Earth System Models (left) and outputs remapped to 0.05° resolution using bilinear (middle) and conservative (right) remapping. Maps were created using R version 4.1.0 (<https://cran.r-project.org/>) ^25^.


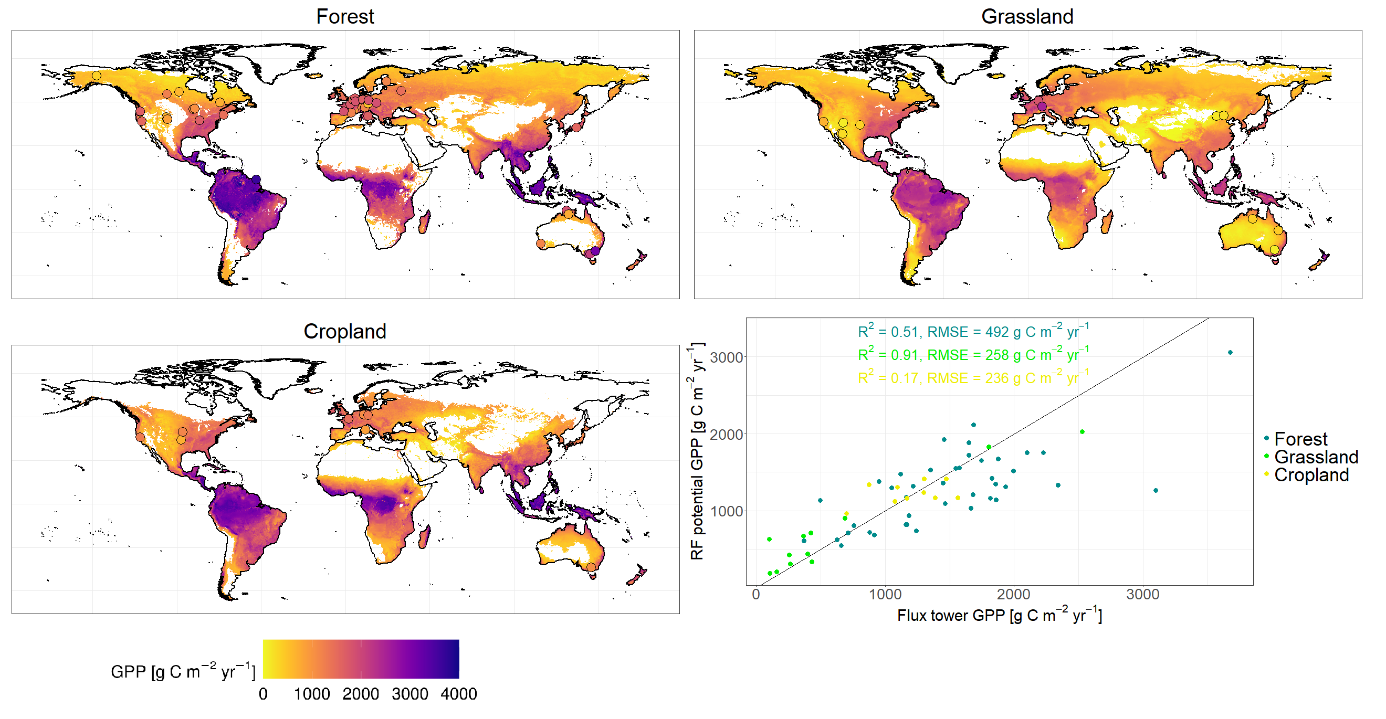


Fig. S16: Comparison of predicted potential GPP to FLUXNET sites (n=63). The figure was created using R version 4.1.0 (<https://cran.r-project.org/>) ^25^.


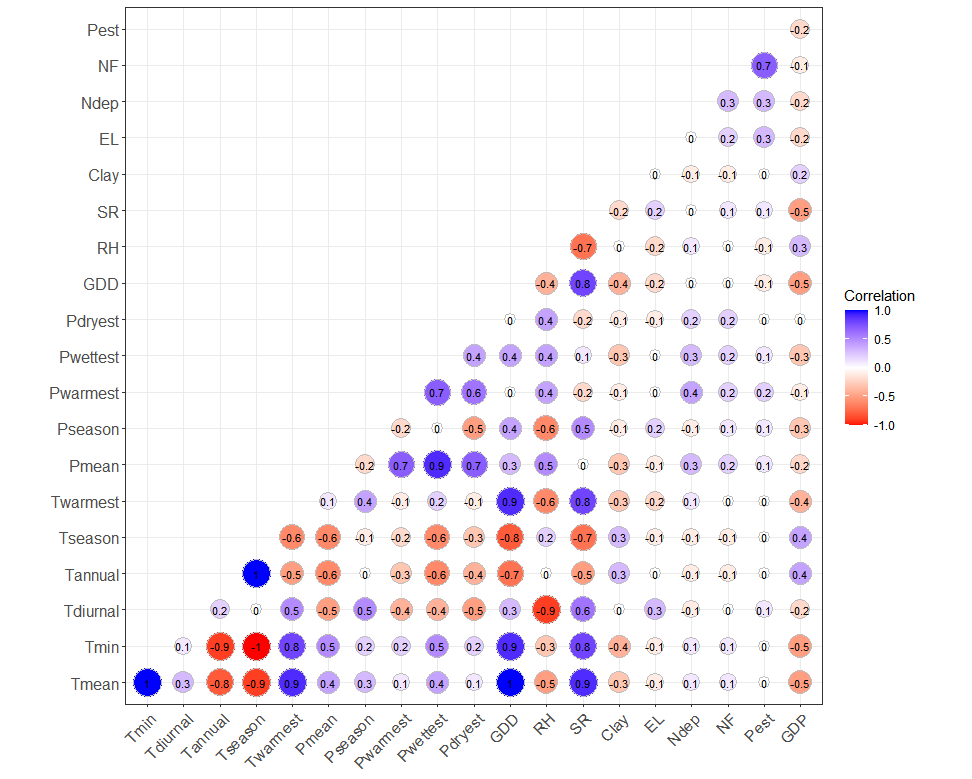


Fig. S17: Correlogram of predictor variables. The figure was created using R version 4.1.0 (<https://cran.r-project.org/>) ^25^.

**References**

1 Li, W. *et al.* Mapping the yields of lignocellulosic bioenergy crops from observations at the global scale. *Earth Syst Sci Data* **12**, 789–804, doi:10.5194/essd-12-789-2020 (2020).

2 Hoffman, A. L., Kemanian, A. R. & Forest, C. E. Analysis of climate signals in the crop yield record of sub-saharan africa. *Global Change Biol* **24**, 143-157, doi:10.1111/gcb.13901 (2018).

3 Sanderman, J., Hengl, T. & Fiske, G. J. Soil carbon debt of 12,000 years of human land use. *P Natl Acad Sci USA* **115**, E1700-E1700, doi:10.1073/pnas.1800925115 (2018).

4 Li, Y. C., Li, M. Y., Li, C. & Liu, Z. Z. Forest aboveground biomass estimation using landsat 8 and sentinel-1a data with machine learning algorithms. *Sci Rep-Uk* **10**, doi:10.1038/s41598-020-67024-3 (2020).

5 Hengl, T. *et al.* Global mapping of potential natural vegetation: An assessment of machine learning algorithms for estimating land potential. *Peerj* **6**, doi:10.7717/peerj.5457 (2018).

6 Parmentier, I. *et al.* Predicting alpha diversity of african rain forests: Models based on climate and satellite-derived data do not perform better than a purely spatial model. *J Biogeogr* **38**, 1164-1176, doi:10.1111/j.1365-2699.2010.02467.x (2011).

7 Ploton, P. *et al.* Spatial validation reveals poor predictive performance of large-scale ecological mapping models. *Nat Commun* **11**, doi:10.1038/s41467-020-18321-y (2020).

8 Li, X. & Xiao, J. F. Mapping photosynthesis solely from solar-induced chlorophyll fluorescence: A global, fine-resolution dataset of gross primary production derived from oco-2. *Remote Sens-Basel* **11**, doi:10.3390/rs11212563 (2019).

9 Zhang, Y. *et al.* Data descriptor: A global moderate resolution dataset of gross primary production of vegetation for 2000-2016. *Sci Data* **4**, doi:10.1038/sdata.2017.165 (2017).

10 Yebra, M., Van Dijk, A. I. J. M., Leuning, R. & Guerschman, J. P. Global vegetation gross primary production estimation using satellite-derived light-use efficiency and canopy conductance. *Remote Sens Environ* **163**, 206-216, doi:10.1016/j.rse.2015.03.016 (2015).

11 Jung, M. *et al.* Scaling carbon fluxes from eddy covariance sites to globe: Synthesis and evaluation of the fluxcom approach. *Biogeosciences* **17**, 1343-1365, doi:10.5194/bg-17-1343-2020 (2020).

12 Tramontana, G. *et al.* Predicting carbon dioxide and energy fluxes across global fluxnet sites with regression algorithms. *Biogeosciences* **13**, 4291-4313, doi:10.5194/bg-13-4291-2016 (2016).

13 Zhao, M. S., Heinsch, F. A., Nemani, R. R. & Running, S. W. Improvements of the modis terrestrial gross and net primary production global data set. *Remote Sens Environ* **95**, 164-176, doi:10.1016/j.rse.2004.12.011 (2005).

14 Pastorello, G. *et al.* The fluxnet2015 dataset and the oneflux processing pipeline for eddy covariance data. *Sci Data* **7**, doi:10.1038/s41597-020-0534-3 (2020).

15 ESA. Land cover cci product user guide version 2. (2017). <<https://www.esa-landcover-cci.org/?q=webfm_send/84>>.

16 Friedl, M. & Sulla-Menashe, D. *Mcd12c1 modis/terra+aqua land cover type yearly l3 global 0.05deg cmg v006*, doi:10.5067/MODIS/MCD12C1.006 (2015).

17 Karger, D. N. *et al.* Data descriptor: Climatologies at high resolution for the earth's land surface areas. *Sci Data* **4**, doi:10.1038/sdata.2017.122 (2017).

18 Karger, D. N. *et al.* *Data from: Climatologies at high resolution for the earth's land surface areas*, doi:10.5061/dryad.kd1d4 (2018).

19 Fick, S. E. & Hijmans, R. J. Worldclim 2: New 1-km spatial resolution climate surfaces for global land areas. *Int J Climatol* **37**, 4302-4315, doi:10.1002/joc.5086 (2017).

20 Wieder, W. R., Boehnert, J., Bonan, G. B. & Langseth, M. *Regridded harmonized world soil database v1.2*, doi:10.3334/ORNLDAAC/1247 (2014).

21 Lamarque, J. F. *et al.* Multi-model mean nitrogen and sulfur deposition from the atmospheric chemistry and climate model intercomparison project (accmip): Evaluation of historical and projected future changes. *Atmos Chem Phys* **13**, 7997-8018, doi:10.5194/acp-13-7997-2013 (2013).

22 Kummu, M., Taka, M. & Guillaume, J. H. A. Data descriptor: Gridded global datasets for gross domestic product and human development index over 1990-2015. *Sci Data* **5**, doi:10.1038/sdata.2018.4 (2018).

23 Hurtt, G. C. *et al.* Harmonization of global land use change and management for the period 850-2100 (luh2) for cmip6. *Geosci Model Dev* **13**, 5425-5464, doi:10.5194/gmd-13-5425-2020 (2020).

24 Eyring, V. *et al.* Overview of the coupled model intercomparison project phase 6 (cmip6) experimental design and organization. *Geosci Model Dev* **9**, 1937-1958, doi:10.5194/gmd-9-1937-2016 (2016).

25 R Core Team. R: A language and environment for statistical computing. R Foundation for Statistical Computing, Vienna, Austria. (2021). <<https://www.R-project.org>>.

26 Meyer, H. & Pebesma, E. Machine learning-based global maps of ecological variables and the challenge of assessing them. *Nat Commun* **13**, doi:10.1038/s41467-022-29838-9 (2022).
